# GWAS meta-analysis identifies five susceptibility loci for endometrial cancer

**DOI:** 10.1016/j.ebiom.2025.105830

**Published:** 2025-07-08

**Authors:** Dhanya Ramachandran, Xuemin Wang, Triin Laisk, Ying Zheng, Nathan Ingold, Daffodil M. Canson, Pik Fang Kho, Bianca J. Naumann, Carly J. Chapman, Kristine Bousset, Anna V. Krause, Peter Schürmann, Britta Wieland, Patricia Hanel, Fabienne Hülse, Norman Häfner, Ingo Runnebaum, Natalia Dubrowinskaja, Nurzhan Turmanov, Tatyana Yugay, Zura Berkutovna Yessimsiitova, Frédéric Amant, Daniela Annibali, Matthias W. Beckmann, Clara Bodelon, Daniel D. Buchanan, Chu Chen, Megan A. Clarke, Linda S. Cook, Immaculata De Vivo, Wout De Wispelaere, Mengmeng Du, Douglas F. Easton, Julius Emons, Peter A. Fasching, Christine M. Friedenreich, Grace Gallagher, Graham G. Giles, Ellen L. Goode, Holly R. Harris, David J. Hunter, David L. Kolin, Peter Kraft, James V. Lacey, Diether Lambrechts, Lingeng Lu, George L. Mutter, Jeffin Naduparambil, Kelli O’Connell, Alpa V. Patel, Paul D.P. Pharoah, Timothy R. Rebbeck, Fulvio Ricceri, Harvey A. Risch, Matthias Ruebner, Carlotta Sacerdote, Rodney J. Scott, V. Wendy Setiawan, Xiao-Ou Shu, Melissa C. Southey, Emma Tham, Ian Tomlinson, Constance Turman, Nicolas Wentzensen, Wanghong Xu, Herbert Yu, Wei Zheng, Amanda B. Spurdle, Yosef Yarden, Estonian Biobank Research Team, Reedik Mägi, Peter Hillemanns, Dylan M. Glubb, Thilo Dörk, Tracy A. O’Mara

**Affiliations:** a Gynaecology Research Unit, Hannover Medical School, Hannover, Germany; b Cancer Research Program, QIMR Berghofer, Brisbane, Queensland, Australia; c Estonian Genome Center, Institute of Genomics, University of Tartu, Tartu, Estonia; d Department of Gynecology, The First Hospital of Hebei Medical University, Shijiazhuang, China; e Population Health Program, QIMR Berghofer, Brisbane, Queensland, Australia; f Department of Gynaecology, Jena University Hospital - Friedrich Schiller University, Jena, Germany; g RU21 GmbH, Jena, Germany; h Clinic for Rheumatology and Immunology, Hannover Medical School, Hannover, Germany; i Rahat Clinics, Almaty, Kazakhstan; j KRH Clinic Neustadt am Rübenberge, Neustadt am Rübenberge, Germany; k Department of Biodiversity and Bioresources, al-Farabi Kazakh National University, Almaty, Kazakhstan; l Division of Gynecologic Oncology, Department of Obstetrics and Gynecology, University Hospitals KU Leuven, University of Leuven, Leuven, Belgium; m Gynecological Oncology Laboratory, Department of Oncology, KU Leuven and Leuven Cancer Institute (LKI), Leuven, Belgium; n Department of Gynecology and Obstetrics, Comprehensive Cancer Center Erlangen-EMN, Friedrich-Alexander University Erlangen-Nuremberg, University Hospital Erlangen, Erlangen, Germany; o Department of Population Science, American Cancer Society, Atlanta, GA, USA; p Colorectal Oncogenomics Group, Department of Clinical Pathology, Melbourne Medical School, The University of Melbourne, Melbourne, Victoria, Australia; q Collaborative Centre for Genomic Cancer Medicine, Victorian Comprehensive Cancer Centre, Parkville, Victoria, Australia; r Epidemiology Program, Fred Hutchinson Cancer Center, Seattle, WA, USA; s Division of Cancer Epidemiology and Genetics, National Cancer Institute, Bethesda, MD, USA; t Epidemiology, School of Public Health, University of Colorado, Aurora, CO, USA; u Community Health Sciences, University of Calgary, Calgary, AB, Canada; v Channing Division of Network Medicine, Department of Medicine, Brigham and Women’s Hospital and Harvard Medical School, Boston, MA, USA; w Department of Epidemiology, Harvard T.H. Chan School of Public Health, Boston, MA, USA; x Unit of Epidemiology, Local Health Unit of Novara, Novara, Italy; y Department of Epidemiology and Biostatistics, Memorial Sloan-Kettering Cancer Center, New York, NY, USA; z Department of Oncology, Centre for Cancer Genetic Epidemiology, University of Cambridge, Cambridge, UK; aa Department of Public Health and Primary Care, Centre for Cancer Genetic Epidemiology, University of Cambridge, Cambridge, UK; ab Department of Cancer Epidemiology and Prevention Research, Alberta Health Services, Calgary, AB, Canada; ac Departments of Oncology and Community Health Sciences, Cumming School of Medicine, University of Calgary, Calgary, AB, Canada; ad Cancer Epidemiology Division, Cancer Council Victoria, Melbourne, Victoria, Australia; ae Centre for Epidemiology and Biostatistics, Melbourne School of Population and Global Health, The University of Melbourne, Melbourne, Victoria, Australia; af Precision Medicine, School of Clinical Sciences at Monash Health, Monash University, Clayton, Victoria, Australia; ag Division of Epidemiology, Department of Quantitative Health Sciences, Mayo Clinic, Rochester, MN, USA; ah Program in Epidemiology, Division of Public Health Sciences, Fred Hutchinson Cancer Center, Seattle, WA, USA; ai Department of Epidemiology, University of Washington, Seattle, WA, USA; aj Nuffield Department of Population Health, University of Oxford, Oxford, UK; ak Brigham and Women’s Hospital, Boston, MA, USA; al Division of Cancer Epidemiology and Genetics, Department of Health and Human Services, National Cancer Institute, National Institutes of Health, Bethesda, MD, USA; am Department of Computational and Quantitative Medicine, City of Hope, Duarte, CA, USA; an City of Hope Comprehensive Cancer Center, City of Hope, Duarte, CA, USA; ao Laboratory for Translational Genetics, Department of Human Genetics, KU Leuven, Leuven, Belgium; ap VIB Center for Cancer Biology, VIB, Leuven, Belgium; aq Chronic Disease Epidemiology, Yale School of Public Health, New Haven, CT, USA; ar Department of Medicine, Brigham and Women’s Hospital, Harvard Medical School, Boston, MA, USA; as Department of Computational Biomedicine, Cedars-Sinai Medical Center, West Hollywood, CA, USA; at Harvard T.H. Chan School of Public Health, Boston, MA, USA; au Dana-Farber Cancer Institute, Boston, MA, USA; av Department of Clinical and Biological Sciences, Centre for Biostatistics, Epidemiology, and Public Health (C-BEPH), University of Turin, Turin, Italy; aw Department of Health Sciences, University of Eastern Piedmont, Novara, Italy; ax Division of Molecular Medicine, Pathology North, John Hunter Hospital, Newcastle, New South Wales, Australia; ay Discipline of Medical Genetics, School of Biomedical Sciences and Pharmacy, Faculty of Health, University of Newcastle, Callaghan, New South Wales, Australia; az Hunter Medical Research Institute, John Hunter Hospital, Newcastle, New South Wales, Australia; ba Department of Preventive Medicine, Keck School of Medicine, University of Southern California, Los Angeles, CA, USA; bb Division of Epidemiology, Department of Medicine, Vanderbilt Epidemiology Center, Vanderbilt-Ingram Cancer Center, Vanderbilt University School of Medicine, Nashville, TN, USA; bc Department of Clinical Pathology, The University of Melbourne, Melbourne, Victoria, Australia; bd Department of Molecular Medicine and Surgery, Karolinska Institutet, Stockholm, Sweden; be Clinical Genetics and Genomics, Karolinska University Hospital, Stockholm, Sweden; bf Department of Oncology, University of Oxford, Oxford, UK; bg School of Public Health, Fudan University, Shanghai, China; bh Epidemiology Program, University of Hawaii Cancer Center, Honolulu, HI, USA; bi Weizmann Institute of Science, Rehovot, Israel; bj School of Biomedical Sciences, Faculty of Medicine, The University of Queensland, Brisbane, Queensland, Australia

**Keywords:** Endometrial carcinoma, GWAS, Luciferase, eQTL, NAV3

## Abstract

**Background:**

Endometrial cancer is the most common gynaecological cancer in high-income countries. In addition to environmental risk factors, genetic predisposition contributes towards endometrial cancer development but is still incompletely defined.

**Methods:**

Building on genome-wide association studies (GWASs) by the Endometrial Cancer Association Consortium, we conducted a GWAS meta-analysis of 17,278 endometrial cancer cases and 289,180 controls, incorporating biobank samples from the UK, Finland, Estonia and Japan.

**Findings:**

GWAS analysis identified five additional risk loci (3p25.2, 3q25.2, 6q22.31, 12q21.2, and 17q24.2). Corresponding gene-based analyses supported findings for three of the five loci, at *NAV3* (12q21.2), *PPARG* (3p25.2), and *BPTF* (17q24.2), as well as two additional candidate risk regions at *ATF7IP2* (16p13.2-p13.13) and *RPP21* (6p22.1). Validation genotyping in further independent case-control series replicated the most significant locus at 12q21.2 and corroborated risk variants located intronic to *NAV3,* the gene for Neuron Navigator 3. Downregulation of *NAV3* in endometrial cell lines accelerated cell division and wound healing capacity whereas *NAV3* overexpression reduced cell survival and increased cell death, indicating that NAV3 acts as a tumour suppressor in endometrial cells.

**Interpretation:**

Our large study extends the number of genome-wide significant risk loci identified for endometrial carcinoma by about one-third and proposes a role of NAV3 as a tumour suppressor in this common cancer.

**Funding:**

This study was mainly supported by funding from the 10.13039/100008672Wilhelm Sander Foundation, Germany, and the 10.13039/501100000925National Health and Medical Research Council (NHMRC) of Australia. A complete list of funding organisations is provided in the acknowledgements.

**Research in context**

**Evidence before this study**

Endometrial carcinoma is a common gynaecological malignancy with a hereditary component. Previous work has uncovered endometrial cancer susceptibility candidate genes, including those responsible for Lynch Syndrome, but also 16 genomic loci harbouring common low-risk variants. Much of this information has been obtained from large case-control studies conducted within two international consortia: the Endometrial Cancer Association Consortium (ECAC) and the Epidemiology of Endometrial Cancer Consortium (E2C2). In addition, national biobanks from the UK, Finland, Japan and Estonia have recently established genomic databases including endometrial cancer cases and controls which had not been fully exploited for endometrial cancer research until now. The present work bases on a combined analysis of these resources to identify novel endometrial cancer risk factors.

**Added value of this study**

We performed the largest endometrial cancer GWAS meta-analysis to date, identifying five previously unreported genomic risk loci, accounting for an additional 1.2% of the familial relative risk of endometrial cancer. Subsequent functional experiments support *NAV3*, that harbours the most strongly associated risk variants, as a tumour suppressor gene in endometrial cells.

**Implications of all the available evidence**

Refining the genetic risk for endometrial cancer helps to improve risk prediction via polygenic risk score calculation, prevention strategies and early interventions. Identifying the underlying molecular mechanisms and cancer drivers may additionally lead to druggable targets and therapeutic avenues.

## Introduction

Cancer of the lining of the *corpus uteri*, also known as endometrial cancer, is a common gynaecological malignancy with more than 410,000 new cases and 97,000 deaths reported worldwide in 2020.^1^^,^^2^ Originating in the inner epithelium of the uterus, about 80% of endometrial cancers belong to the oestrogen-dependent endometrioid histological subtype that generally have good prognosis, with more aggressive non-endometrioid subtypes making up the remainder of cases.^3^, ^4^, ^5^ Endometrial cancer is detected mostly in older women, and age- and treatment-related comorbidities may result in higher fatalities.^6^ However, incidence is rising in younger women and, unlike most other tumour types, mortality is rising in developed countries.^7^ Known risk factors are nulliparity, obesity, and oestrogen exposure unopposed by progesterone.^1^^,^^8^, ^9^, ^10^ Germline genetic variation also contributes to endometrial cancer risk.^11^^,^^12^ While some of the genetic risk can be attributed to cancer predisposition syndromes such as Lynch syndrome (hereditary non-polyposis colorectal cancer) and Cowden syndrome (part of the PTEN hamartoma tumour syndrome),^13^, ^14^, ^15^, ^16^, ^17^ a large portion of genetic heritability remains unexplained.^18^^,^^19^ Genome-wide association studies of endometrial cancer led by the Endometrial Cancer Association Consortium (ECAC) have been pivotal in identifying common genetic risk variants, with 16 risk loci supported by robust evidence,^20^, ^21^, ^22^, ^23^ while multi-trait analyses identified six further candidate susceptibility loci for endometrial cancer.^24^, ^25^, ^26^, ^27^ Biobanking initiatives such as those from the UK, Finland, Japan and Estonia, although often individually underpowered for detecting endometrial cancer GWAS risk loci,^28^, ^29^, ^30^, ^31^ can act as valuable resources for acquiring additional samples for GWAS meta-analysis. We hypothesised that an endometrial cancer GWAS meta-analysis incorporating ECAC and biobank samples may reveal additional GWAS risk loci due to increased power. Thus, in this study we report on the largest endometrial cancer GWAS meta-analysis to date, including 17,278 endometrial cancer cases and 289,180 controls, followed by replication genotyping in two further case-control cohorts.

## Methods

### GWAS meta-analysis

**Fig. 1:**
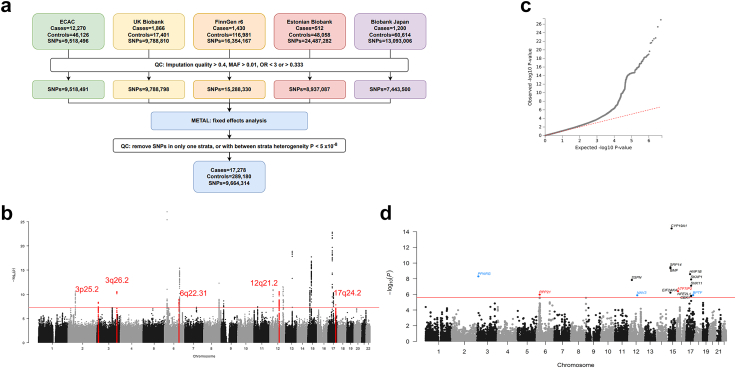
**GWAS meta-analysis for endometrial cancer.** (a) Workflow showing the different studies that contributed summary statistics and QC steps involved, (b) Manhattan plot displaying chromosomes on the x-axis in alternating black and light grey tones, with -log_10_ p values after meta-analysis on the y-axis, genome-wide significance threshold is set at 5 × 10^−8^ in a red line, and variants underlying the five genomic loci identified in this study are coloured as red dots, (c) Quantile-quantile plot of expected versus observed p values from the GWAS summary statistics, (d) Manhattan plot after genome-wide gene-based MAGMA analysis displays chromosomes on the x-axis in alternating black and light grey hues, with -log_10_ p values on the y-axis. Genome-wide significance threshold is set to 2.45 × 10^−6^ and all significant genes above this threshold are labelled, with the gene names coloured in red and blue. Loci that only come up in the MAGMA analysis are labelled in red whereas loci that were identified in both the meta-analysis and MAGMA analysis are in blue.

In order to perform a meta-analysis, GWAS summary statistics were extracted for Endometrial cancer versus controls from ECAC, UK Biobank (UKB), Biobank Japan (BBJ), Estonian Biobank (EstBB) and FinnGen (Release 6, https://r6.finngen.fi/pheno/C3_CORPUS_UTERI_EXALLC) cohorts as shown in Supplementary Table S1. A total of 17,278 endometrial cancer cases and 289,180 cancer-free controls were meta-analysed in the current study (Fig. 1a). The FinnGen dataset was harmonised to the human genome build GRCh37 with UCSC LiftOver for compatibility with the other datasets. Variants with imputation quality more than 0.4, minor allele frequency (MAF) > 0.01, and ORs < 3 or >0.333 were selected.

Participant characteristics for the FinnGen data release R6, for this GWAS dataset, are available at https://r6.risteys.finngen.fi/phenocode/C3_CORPUS_UTERI with mean age at first event reported to be 64.06 years for the 1430 cases. For all participants, regardless of sex or phenotype, a median age of 63 years was reported.^30^ FinnGen has been noted to be not epidemiologically representative due to its open patient recruitment measures, however, this is not expected to cause any GWAS biases.^30^

For the GWAS dataset arising from the Biobank Japan cohort, detailed information has been provided previously,^32^, ^33^, ^34^ with mean age of female participants in the “uterine corpus cancer” cohort reported to be 58.91 years, and mean age of all females in the biobank reported to be 61.5 years. While survival rates have been similar to the whole population in the Japanese biobank,^33^ this cohort involves individuals with diseases and is not tested against the general population.

For the ECAC GWAS study, QC and GWAS details have been reported,^22^ with imputation using the 1000 Genomes Project v3 reference panel, association testing via logistic regression, with PC adjustment. The average age ( ± S.D.) of cases was 53.4 y ± 13.4 y and of controls was 63.1 y ± 9.1 y. The samples collected by the ECAC are considered to be representative for the respective countries as the recruitment was not restricted to familial or early-onset cases.

As reported previously,^24^ the UK Biobank endometrial cancer GWAS was performed (application number 25331), with details on genotyping and imputation for the dataset provided.^35^ Cases were defined by ICD10 code (C54) from hospital and cancer registry records (data codes: 400006, 41270 and 41202) with randomly selected female cancer-free controls. Controls were excluded if they had previously undergone a hysterectomy. The GWAS was performed using REGENIE^36^ via a logistic mixed model, after adjustment for the genotyping array and the top 10 PCs, followed by QC. The average age (± S.D.) of cases was 60.8 y ± 6.2 y and of controls was 55.9 y ± 8 y.

The Estonian Biobank GWAS data for endometrial cancer have not been reported previously and were derived from a population-based biobank which can be considered representative of the population.^31^ Details on design and population recruitment have been described previously^31^ and data from the 200 K data freeze were used for the analysis. Information on ICD10 codes was obtained via regular linking with national registries. ICD code C54.1 Malignant neoplasm: Endometrium were taken as cases, with cancer-free females (no relevant C- or D-codes) used as controls. Here, the average age (± S.D.) of cases was 63.1 y ± 11.6 y and of controls was 41.7 y ± 16.7 y. Estonian Biobank samples were genotyped in Core Genotyping Lab of the Institute of Genomics, University of Tartu using Illumina GSAv1.0, GSAv2.0, and GSAv2.0_EST arrays. Individuals were excluded from the analysis if their call-rate was <95% or sex defined based on heterozygosity of X chromosome did not match sex in phenotype data. Variants were filtered by call-rate <95% and HWE p value < 1 × 10^−4^ (autosomal variants only). Variant positions were updated to b37 and all variants were changed to be from TOP strand using tools and reference files provided in https://www.well.ox.ac.uk/∼wrayner/strand/. Before imputation, variants with MAF < 1% and indels were removed. Pre-phasing was done using Eagle v2.3^37^ software (number of conditioning haplotypes Eagle2 uses when phasing each sample was set to: --Kpbwt = 20000) and imputation was done using Beagle v.28Sep18.793^38^ with effective population size ne = 20,000. Estonian population specific imputation reference of 2297 WGS samples were used.^39^ All samples included in the GWAS were of European ethnicity. GWAS testing was carried out using SAIGE v 0.43.1 without the LOCO option, using filter --minMAC = 5 in the association testing stage. The following association testing model was used: diagnosis ∼ Variant + Age_at_recruitment together with 10 principal component (PCs). The genomic inflation factor (λ) was 0.89 with lower frequency variants and rare variants accounting for the deflation, together with smaller case count.

Given known estimates, the majority of the samples (∼80%) are likely of endometrioid origin.^40^ While this information is not available for the Biobank data, the ECAC study includes around 67% cases of the endometrioid type.^22^

Meta-analysis was performed using the fixed effects inverse-variance weighted method implemented in METAL^41^ (version released on 2011-03-25). Variants assessed in at least two studies and with between strata heterogeneity p > 5 × 10^−8^ were retained for further analysis, leading to a total of 9,664,314 SNPs. To identify independent risk loci, we performed approximate conditional analyses (COJO) implemented in GCTA.^42^^,^^43^ Using the conventional genome-wide significance threshold, 5 × 10^−8^, all variants located within ± 500 kb of a significant SNP were initially considered as part of that locus. A panel of 10,000 unrelated individuals randomly selected from UKBB^44^ were used as the LD reference in GCTA-COJO. We used a BFDP test^45^ to assess the probability of a variant being a false positive, based on the p value and an upper likely OR of 1.2 using the genetic analysis package GAP^46^ in R v4.2.0. BFDP tests were performed using two priors: 1:10,000 and 1:1000. GWAS variants were visualised and 95% credible risk variant sets identified by Bayesian refinement methods within the LocusZoom platform^47^^,^^48^ (Supplementary Table S2). Briefly, the posterior probability of being casual (PPi) was calculated for each variant in the region. Variants were sorted by PPi in descending order and added to the credible set until the sum of PPi reached ≥ 95%. The contribution of risk variants to familial relative risk of endometrial cancer was calculated using the formula detailed in Eeles et al.^49^ The familial relative risk was assumed to be 2-fold, as per that reported by observational studies.^12^^,^^19^ We also assumed that the loci had log–additive association with risk and were not in LD.

### FUMA webtool: MAGMA gene-based analysis

The GWAS meta-analysis summary statistics was submitted to FUMA v1.3.7 (Job ID: 175147). The genes mapped to SNPs were taken for MAGMA gene-based analyses. Whole genome gene-based analyses were performed via MAGMA v1.08, with SNPs within a 25 kbp window mapped to genes. In total, 20,174 protein coding genes were used to identify genome-wide significant genes, setting the GWS threshold for this analysis at p = 0.05/20174 = 2.45 × 10^−6^.

### Patients

In order to perform replication genotyping, EDTA whole blood was available from 411 patients with endometrial cancer from Germany (241 from Hannover Medical School and 170 from the Friedrich Schiller University of Jena), and from 1122 cancer-free controls (Hannover Medical School). For the German endometrial cancer case-control cohort, the mean age (range) of cases and controls was 64.4 (29–89) and 33.4 (18–68) years, respectively. The German case cohort consisted of 241 patients from Lower Saxony and 170 patients from Thuringia. Controls were healthy female blood donors from the Transfusion Department at Hannover Medical School (Lower Saxony). All patients and controls were of German descent (Supplementary Table S3).

A second cohort of 479 patients with endometrial cancer was available from Almaty University in Kazakhstan.^50^ For this second cohort, patient enrolment occurred between the years of 2001–2010, with 122 patients of Asian (mainly Altaic) and 307 patients with European (mainly Russian) ancestry with median age at diagnosis of 60 years (range 32–84 years) for endometrial cancer. A set of 660 cancer-free controls from Kazakhstan were also at hand (498 with Asian and 84 with European ancestry), with age being 38.8 years (range 18–71 years) for controls. The cases and controls in the European Kazakh Meta Population were mostly Russians, and the Asian Kazakh Meta Population consisted largely of Kazakhs (Supplementary Table S3). Written, informed consent was taken from all patients and DNA was extracted at Hannover Medical School using standard phenol chloroform extraction, with concentration checked using a Nanodrop8000 spectrophotometer.

We only included cases with a histologically confirmed endometrial carcinoma and had few documentation gaps for the age at first diagnosis: in the German series, this variable remained incompletely defined for 6/411 cases (1.4%), and in the Kazakh series for 9/429 cases (2.1%).

These samples were collected in a hospital-based approach at the respective oncology centres in Hannover, Jena, and Almaty, not through specialised family counselling centres. There were no preferences or restrictions regarding family history of cancer or early age at diagnosis, and most of the patients were incident cases at the time of recruitment, so we consider them representative of the population. The median age at diagnosis of 65 years for our German endometrial cancer series was only slightly lower when compared to the nation-wide median age at diagnosis of endometrial cancer of 67 years documented by the German Cancer Registry at the Robert Koch Institute (https://doi.org/10.18444/5.03.01.0005.0018.0002). The median and mean age at diagnosis of 60 years for our Kazakh endometrial cancer series matches the average age of 60.4 years reported for uterine cancer in Kazakhstan.^51^

### Ethics approval and consent to participate

Informed consent was obtained from all participants according to the Helsinki declaration. This study was approved by the Ethics committee at Hannover Medical School (Vote no. 5833). All Estonian Biobank participants have signed a broad informed consent for using their data in research and the study was carried out under ethical approval 1.1–12/624 from the Estonian Committee on Bioethics and Human Research (Estonian Ministry of Social Affairs) and data release 6–7/GI/18891 from the EstBB.

### Wet-lab genotyping

We selected variants for replication genotyping based on LDlink 1000G EUR threshold R2 0.3 at each of the five previously unreported loci. Fluidigm SNPtype genotyping was performed in the German and Kazakh cohorts for variants listed under Supplementary Table S4 as per manufacturer’s instructions on a Biomark HD system, taking along two non-template controls as negative controls per run. Fluidigm assay IDs per variant with the alleles tested and number of samples genotyped per SNP are listed in Supplementary Table S2. One SNP on chromosome 12, rs1382638, was genotyped using a TaqMan assay, as per manufacturer’s instructions (Supplementary Table S5). At least 10 percent of the samples were checked for concordance with repeated genotyping. The variants were checked for clustering after genotyping (Supplementary Fig. S1) and tested for fitting Hardy Weinberg equilibrium (https://wpcalc.com/en/equilibrium-hardy-weinberg/). Variants were tested for association with case-control status in logistic regression analysis using STATA v17, for each of the individual cohorts. To avoid confounding due to population stratification in the Kazakh case-control series, European and Altaic subpopulations were grouped based on the clinically documented ethnicity of the participants and were separately analysed. A meta-analysis was performed using STATA v17 and forest plots were made for visualization.

### Integration of GWAS and functional genomic data

Previously identified H3K27Ac HiChIP chromatin loops in normal immortalised and tumoural (E6E7hTERT, ARK-1, Ishikawa, and JHUEM-14) cell lines, intersecting with gene promoters, were taken for the integration of GWAS and functional genomic data.^52^ These loops were intersected with credible risk variants to identify candidate target genes. The assessment of gene expression associations with credible risk variants in tissues relevant to endometrial cancer development, including subcutaneous adipose, visceral adipose, whole blood, fibroblasts, EBV-transformed lymphocytes, ovary, and uterus was assessed using the QTLbase2^53^ database (http://mulinlab.org/qtlbase), which also included data for endometrium-specific eQTLs.^54^ Variants were also looked up to be cis-eQTLs in the FIVEx eQTL browser.^55^

### Cell culture and transfection

Ishikawa cells (RRID:CVCL_2529) were cultured in Eagle’s MEM medium supplemented with 5% foetal calf serum and 1% Penicillin-Streptomycin solution and 1% L-Glutamine (Biowest) whereas E6E7hTERT endometrial cells^52^^,^^56^ were cultured using DMEM (Gibco) supplemented with 10% foetal calf serum and 1% Penicillin-Streptomycin solution and 1% L-Glutamine at 5% CO_2_ and trypsinised using 1 × Trypsin-EDTA (Biowest). For seeding, cells were counted using Trypan blue dye on a Neubauer chamber. Cell line authentication was performed by STR profiling. Both cell lines were negative for Mycoplasma contamination in a PCR based test.

### Reporter gene analysis

A *NAV3* promoter luciferase reporter construct was generated by inserting 2215 bp of synthesised DNA (Integrated DNA Technologies, Singapore), containing a *NAV3* transcription start site (chr12:78511404-78513618; GRCh37), into the *Kpn*I and *Hin*dIII sites of pGL3-Basic (Promega Australia). A region encompassing rs1842126 (chr12:78333615-78334114) was synthesised, including both allelic variants, and inserted into *Bam*HI and *Sal*I sites downstream of the Firefly luciferase gene in the *NAV3* promoter construct. E6E7hTERT and Ishikawa were transfected with equimolar amounts of luciferase reporter constructs and 50 ng of the *Renilla* luciferase pRL-SV40 construct with Lipofectamine 2000. The total amount of transfected DNA was maintained at 600 ng for each construct by adding pUC19 (RRID: Addgene_50005) as a carrier plasmid. Luciferase activity was measured 24 h post-transfection using the Dual-Glo Luciferase Assay System (Promega Australia). To correct for variation in transfection efficiency or cell lysate preparation, Firefly luciferase activity was normalised to that of the *Renilla* luciferase. Data were log-transformed and statistical significance was assessed by two-way ANOVA, followed by Dunnett’s multiple comparisons test in GraphPad Prism (version 7.02, GraphPad Software, San Diego, CA, USA).

### NAV3 silencing and overexpression

Lipofectamine 2000 was used to transfect E6E7hTERT cells whereas Lipofectamine 3000 was used for Ishikawa cells as per manufacturer’s instructions (Thermo Fisher Scientific). An ON-TARGETplus™ siRNA smartpool from Dharmacon was used for silencing *NAV3* in both cell lines (final concentration of 100 pmol in Ishikawa and 200 pmol in E6E7hTERT cells), along with siGENOME non-targeting siRNA pool #2 as negative control. This was followed by Trizol based RNA isolation, cDNA synthesis, and qRT-PCR in technical triplicates to confirm gene silencing using PrimeTime™ qPCR Probe assays and gene expression master mix (IDT), taking *RPL13A* as housekeeper (all sequences provided in Supplementary Table S6).

We obtained pEGFPN1, pEGFPN1-NAV3 (wildtype) and pEGFPN1-NAV3D1047N (mutant) plasmids^57^ for overexpression studies of NAV3 in Ishikawa cells. Endotoxin-free plasmids were grown in 10-beta competent *E. coli* cells (NEB) and the isolated plasmid DNA (Macherey–Nagel midi isolation kit) was quality checked on a Nanodrop8000 instrument. Plasmid sequences were confirmed by next generation sequencing (Azenta Biosciences) (Supplementary Fig. S2).

The pEGFPN1-NAV3D1047N construct acted as a negative control for the wildtype expressing pEGFPN1-NAV3WT vector. All plasmids were transfected into Ishikawa cells using Fugene4K (Promega) by mixing 6:1 with 1 μg of plasmid DNA as per manufacturer’s instructions. Successfully transfected green-fluorescing cells were visualised after 24 h on a Leica DMI6000B microscope or on a Keyence BZ-X800 system. Overexpression was also confirmed by Trizol based RNA isolation, cDNA synthesis, and qRT-PCR in technical triplicates and by immunoblotting.

### Immunoblotting and immunocytochemistry

Cells after silencing or overexpression were lysed using an extraction buffer (50 mM Tris pH 7.4, 150 mM NaCl, 2 mM EGTA, 2 mM EDTA, 25 mM NaF, 0.1 mM Na_3_VO_4_, 0.1 μM PMSF, 25 mM β-glycerophosphate, 5 μg/ml Leupeptin, 1 μg/ml Aprotinin, 0.2% Triton X-100, and 0.3% NonidetP-40) for 30 min on ice to obtain protein lysates. The lysates were quantified on a UV Photometer (Eppendorf) using Protein Assay Dye Reagent Concentrate (Bio-Rad), prepared for loading using a buffer (0.5 M Tris HCl pH 6.8, 10% SDS, Glycerol, β-Mercaptoethanol, 1 mg/ml Bromophenol blue), and heated at 95 °C for 5 min. 20–50 μg lysate was loaded onto a 5.1% stacking gel at 0.5 M Tris HCl pH 6.8 and then separated through 4.5% SDS-PAGE at 1 M Tris HCl pH 8.7, using the Precision plus protein all blue protein standard marker (BioRad) along with lysates from HCT116 p53 WT or HCT116 p53 −/− cells as positive controls for NAV3 expression. Proteins were transferred to a nitrocellulose membrane and incubated with 1:250 Rabbit anti-NAV3 (Sigma–Aldrich HPA032111/HPA032112, RRID:AB_10603881), followed by incubation with 1:3000 ECL linked F(ab′)2 fragments from donkey anti-Rabbit IgG HRP (GE Healthcare NA9310, RRID:AB_772193). For loading control, the membranes were incubated with 1:600 mouse anti-PRKDC (catalytic subunit of DNA-PK, Calbiochem NA57), followed by incubation with 1:3000 ECL linked F(ab′)2 fragments from donkey anti-mouse IgG HRP (GE Healthcare NA9340). For detection, a horseradish peroxidase (HRP) based enhanced chemiluminescence detecting enzyme was used (Western Bright systems).

For immunocytochemistry, 20,000 Ishikawa cells or 15,000 E6E7hTERT cells were seeded on sterile coverslips (Menzel) in 24 well plates and treated with siRNA after 24 h. After 72 or 48 h, respectively, the coverslips were washed with 1 × PBS, fixed with cold Methanol, and lysed with 0.2% Tween-20 in 1 × PBS. After washing, the coverslips were incubated in primary antibody (Rabbit anti-NAV3 Sigma–Aldrich HPA032111, RRID:AB_10603881) diluted 1:350 in 2% normal goat serum (NGS) in 1 × PBS for 1 h 30 min at room temperature. After washing, the prepared secondary antibody, Alexa-Fluor 546 goat anti-rabbit (Invitrogen, RRID:AB_143051) 1:250 in 2% NGS in 1 × PBS, was added onto the coverslip for 45 min at room temperature. After washing, 0.3 uM DAPI (Invitrogen) in 1 × PBS was added onto the coverslips for 10 min. The coverslips were then mounted immediately onto 10 μl of pro Long Gold Antifade (Invitrogen) on glass slides (Menzel). The slides were imaged the next day on a Keyence BZ-X800 microscope at 40× magnification (blue channel—DAPI, red channel—anti-NAV3, green channel—GFP fluorescence). Antibody HPA032111 has been validated by the Human Protein Atlas (HPA) project, and NA57 was validated for use as noted on the manufacturer’s website.

### Mitotic timing and survival assay

After gene silencing, cells were visualised for up to 72 h on a Leica DMI6000B microscope with incubator BL (Leica) at 37 °C, with 5% CO_2_ supply, with phase contrast images taken in at least 6 technical replicates per well, at 15-min intervals. Cells undergoing mitosis were identified through visual inspection of the movies, and mitotic start and end times were noted down to calculate the time spent in mitosis.^58^ The duration of mitosis was plotted in a Kaplan–Meier (KM) plot using GraphPad Prism v10.

After overexpressing the EGFPN1, EGFPN1-NAV3WT or EGFPN1-NAV3D1047N plasmids, only cells with green fluorescence were visualised for up to 96 h on the Leica DMI6000B system (bright field—for visualizing cells, and green channel—for GFP fluorescence), noting the time from their first detection until their end point (two categories: cell death (1), and mitosis or continuing to be green without any morphological change (0)). The proportion of dying cells over time was plotted in a survival plot using GraphPad Prism v10.

### Scratch assay

Cells were treated with siRNA and after 48 h were transferred onto a 12-well plate coated with 0.2% Gelatin (Sigma). After 24 h, a scratch was drawn in two technical replicates and images were taken using an Olympus SC50 CKX53 microscope at 0, 24, 48 and 72 h. The area of the scratch was analysed using ImageJ and normalised to the value at 0 h. The percentage reduction in scratch area over time was plotted on GraphPad Prism v10 to compare treatment versus control.

### Flow cytometry based live dead analysis

Cell supernatant and trypsinised cells were collected after 72 h after silencing for Ishikawa cells and 48 h for E6E7hTERT cells, and stained with 5 μg/ml Propidium Iodide. The cells were immediately analysed on MACSQuant VYB flow cytometer (Miltenyi) and 20,000 events per treatment were recorded. Cells positive for PI (dead cells) were gated using FlowJo v10 software to calculate percentage of death, these numbers were visualised on GraphPad Prism v10, followed by statistical analysis.

For overexpression experiments in Ishikawa cells after 24 h, cell supernatant and live cells were harvested and stained with 5 μg/ml DAPI and analysed on the MACSQuant VYB flow cytometer (Miltenyi) (we used DAPI here because Propidium Iodide and the GFP from the fusion proteins had similar excitation wavelengths). Cells positive for DAPI (dead cells) were visualised on FlowJo v10 software and numbers were taken to calculate percentage of death.

### xCELLigence impedance measurement

Impedance was measured in 96-well E-View plates on an RTCA system (Roche) in Ishikawa and E6E7hTERT cells in technical quintuplicates after silencing treatment. Cell index values were normalised to 2 h after treatment and plotted as 24, 48, 72, and 84 h values in GraphPad Prism v10. Negative siRNA versus *NAV3* silenced cells were compared across timepoints in ANOVA.

### Flow cytometry-based cell cycle analysis

After treatment, cells were harvested using trypsin-EDTA as usual and fixed using cold 70% ethanol at 4° C, followed by RNase treatment and staining with Propidium Iodide. Flow cytometry was performed on a MACSQuant system (Miltenyi) and cell cycle analysis was performed using FlowJo v10.

### ATP assay

After treatment in 96 well plates, CellTiter-Glo® 2.0 Cell Viability Assay (Promega) was used to determine amount of ATP in the cells in technical triplicates as per manufacturer’s instructions. A standard curve was determined using serial dilutions of 100 mM rATP (Promega) in each experiment. The luminescence for all samples was recorded using the Fluoroskan Ascent microplate reader (Thermoscientific).

### Statistical analysis

For GWAS meta-analysis, the standard genome-wide significance threshold of 5 × 10^−8^ was taken. For the replication phase, a p value < 0.05 with an effect direction matching the GWAS meta-analysis was taken as supportive evidence after logistic regression analysis in STATA 17. For functional experimental data, two groups were tested using Student’s t-test (paired or unpaired, as specified in the figure legends), and more than two groups were tested using ANOVA in GraphPad Prism v10. Where applicable, two-way or three-way ANOVA was performed to include experiment as covariable. Biological replicates were used for statistical tests as indicated.

### Role of funders

The funders had no role on experimental design, data acquisition, analysis or interpretation.

## Results

We performed fixed-effects GWAS meta-analyses for 17,278 females with endometrial cancer and 289,180 cancer-free females by combining summary data from the Endometrial Cancer Association Consortium, UK Biobank, FinnGen Biobank, Estonian Biobank and Biobank Japan^28^, ^29^, ^30^, ^31^ (Fig. 1a, with study specifics provided in Supplementary Table S1). We detected five loci (3p25.2, 3q26.2, 6q22.31, 12q21.2, and 17q24.2) and confirmed 14 known loci at genome-wide significance (GWS, p < 5 × 10^−8^) (Fig. 1b, Table 1, forest plots in Supplementary Fig. S3). We also performed random effects meta-analysis for the five top genetic loci, finding little to no difference to the estimates generated through the fixed-effects analysis. The Bayesian false-discovery probability (BFDP) for all loci was <5% assuming a maximum likely odds ratio (OR) of 1.2 and a prior of 1:10,000 (Table 1). An additional known locus at 17q11.2 had a BFDP of 3.6% using a 1:1000 prior. We additionally identified independent signals at two known loci, 8q24.21 and 2p16 (Table 1). Twenty candidate loci with p < 10^−6^ were identified, of which 10 had a BFDP <15% using a 1:10,000 prior (Supplementary Table S7). The corresponding quantile-quantile plot of p values is shown in Fig. 1c and the genomic inflation factor (λ_1000_) was calculated to be 1.003. Independent risk loci from COJO analysis are noted in Supplementary Table S8. We further performed gene-based analyses in MAGMA,^59^ providing evidence of association for genes at three of the five previously unreported GWAS loci (*PPARG* at 3p25.2, *NAV3* at 12q21.2 and *BPTF* at 17q24.2) and 10 previously identified GWAS risk loci. We identified two additional candidate risk regions at *ATF7IP2* (16p13.2-p13.13) and *RPP21* (6p22.1) through gene-based analysis that were not found by the single variant GWAS (Fig. 1d, Supplementary Table S9).

**Table 1:** Endometrial cancer GWAS meta-analysis results for novel genome-wide significant loci and known loci.

Locus	MarkerName	chr:pos (b37)	EA	OA	EAF^a^	FreqSE^a^	MinFreq^a^	MaxFreq^a^	OR (95% CI)	p-value	*I*^*2*^	P_het_	BFDP (%)^b^	
													1:10000	1:1000
Novel loci														
3p25.2	rs11716748	3:12336399	A	G	0.71	0.07	0.53	0.76	1.09 (1.06–1.12)	4.38E-09	0	0.92	<1	<1
3q26.2	rs4459939	3:168643983	T	C	0.26	0.05	0.19	0.40	1.10 (1.07–1.13)	2.80E-11	0	0.46	<1	<1
6q22.31	rs34283499	6:122402574	T	C	0.34	0.02	0.28	0.35	0.92 (0.90–0.95)	1.17E-09	0	0.90	<1	<1
12q21.2	rs1677893	12:78338386	A	T	0.46	0.04	0.35	0.53	1.09 (1.06–1.12)	2.91E-11	0	0.49	<1	<1
17q24.2	rs5024718	17:65892587	T	C	0.69	0.15	0.17	0.75	0.91 (0.88–0.94)	1.59E-08	0	0.89	1.1	<1
Known loci														
2p16.1^c^	rs7579014	2:60707894	A	G	0.63	0.06	0.02	0.65	1.09 (1.12–1.06)	6.85E-10	0	0.42	<1	<1
2p16.1^c^	rs148261157	2:60897579	A	G	0.04	0.00	0.03	0.04	1.27 (1.18–1.36)	3.21E-11	0	0.91	<1	<1
6p22.3	rs1740828	6:21649085	A	G	0.47	0.07	0.22	0.53	0.86 (0.84–0.88)	9.49E-28	47.8	0.11	<1	<1
6q22.31	rs2747716	6:126008372	A	G	0.60	0.06	0.57	0.84	1.11 (1.08–1.14)	3.97E-16	0	0.74	<1	<1
8q24.21^d^	rs10505508	8:129215924	T	C	0.32	0.02	0.30	0.41	0.92 (0.89–0.94)	4.78E-09	0	0.98	<1	<1
8q24.21^d^	rs4733613	8:129599278	C	G	0.14	0.01	0.12	0.15	1.15 (1.11–1.19)	5.27E-13	46	0.14	<1	<1
8q24.21^d^	rs139584729	8:129623902	C	G	0.98	0.00	0.98	0.99	1.39 (1.25–1.55)	2.59E-09	0	0.94	3.8	<1
9p21.3	rs1590625	9:22259421	A	G	0.06	0.00	0.05	0.06	1.17 (1.23–1.11)	2.56E-09	15.1	0.32	<1	<1
11p13	rs3858458	11:32484594	T	C	0.37	0.00	0.36	0.38	1.09 (1.12–1.05)	3.88E-08	27.7	0.24	<1	<1
12p12.1	rs7959150	12:26428063	A	G	0.25	0.01	0.23	0.26	0.91 (0.93–0.88)	1.22E-11	11.8	0.34	<1	<1
12q24.12	rs7310615	12:111865049	C	G	0.47	0.02	0.42	0.48	0.91 (0.94–0.89)	2.98E-12	0	0.90	<1	<1
12q24.21	rs10850382	12:115214548	T	C	0.30	0.05	0.09	0.32	1.09 (1.06–1.12)	2.55E-09	0	0.76	<1	<1
13q22.1	rs9600103	13:73811879	A	T	0.74	0.03	0.72	0.82	1.15 (1.12–1.19)	1.45E-19	0	0.44	<1	<1
15q15.1	rs998713	15:40378467	A	G	0.61	0.04	0.59	0.74	0.91 (0.94–0.89)	4.18E-12	0	0.96	<1	<1
15q21.2	rs17601876	15:51553909	A	G	0.50	0.05	0.34	0.52	0.89 (0.87–0.92)	2.06E-18	0	0.98	<1	<1
17q12	rs11263763	17:36103565	A	G	0.57	0.05	0.53	0.68	1.14 (1.17–1.11)	1.65E-23	0	0.80	<1	<1
17q21.32	rs1860862	17:46204836	A	C	0.39	0.00	0.39	0.41	0.91 (0.94–0.89)	4.81E-11	0	0.95	<1	<1
Known loci not observed at GWS in this meta-analysis														
1p34.3	rs113998067	1:38073356	T	C	0.95	0.01	0.95	0.98	0.86 (0.81–0.92)	1.01E-05	79.8	0.00	84	35
8q24.21	rs35286446	8:129445863	G	GAT	0.41	0.02	0.34	0.42	0.94 (0.92–0.97)	6.79E-06	40.6	0.17	77	25
14q32.33	rs2498796	14:105243220	A	G	0.33	0.07	0.30	0.55	1.05 (1.02–1.08)	8.22E-04	57.8	0.05	100	96
17q11.2	rs1129506	17:29646032	A	G	0.61	0.01	0.59	0.62	0.93 (0.91–0.96)	6.67E-07	41.4	0.16	27	3.6

EA: effect allele; OA: other allele; EAF: effect allele frequency; OR: odds ratio; CI: confidence interval; *I*^*2*^: measurement of heterogeneity (0–100%); P_het_: p value for heterogeneity; BFDP: Bayes false discovery probability.

a Weighted frequency of effect allele across all studies, associated standard error (FreqSE) and minimum/maximum effect allele frequency observed across all studies (MinFreq/MaxFreq).

b BFDP was calculated using an odds ratio of 1.2 and a prior probability of either 1:10000 or 1:1000.

c Results from 2p16.1 represent two independent signals. The r2 between rs7579014 and rs148261157 is 0.0027.

d Results from 8q24.21 represent three independent signals. The r2 between rs10505508 and rs4733613 is 0.003; r2 = 0.0016 between rs10505508 and rs139584729; r2 = 0.0024 between rs4733613 and rs139584729.

**Fig. 2:**
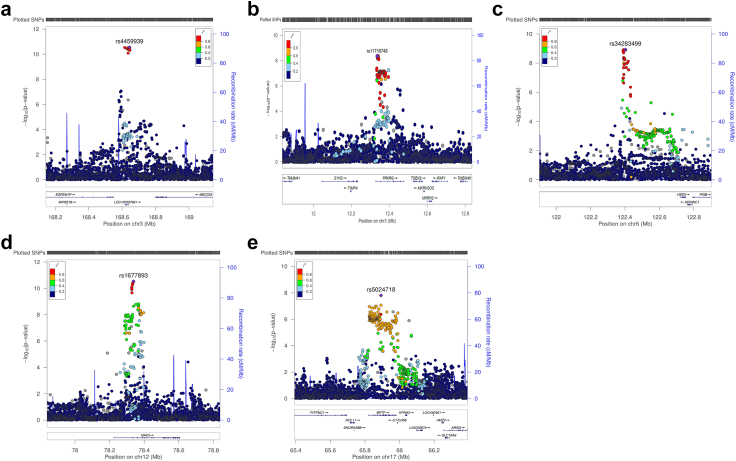
**Five genomic loci for endometrial cancer.** LocusZoom plots at (a) 3p25.2, (b) 3q26.2, (c) 6q22.31, (d) 12q21.2, and (e) 17q24.2 show chromosomal position on the x-axis, genes located within 1 Mbp of the lead SNP, −log_10_ p values on the left y-axis, recombination rate on the right y-axis, and linkage with the lead SNP is colour coded according to the legend (r^2^ ≥ 0.8 in red, 0.6–08 in yellow, 0.4–0.6 in green, 0.2–0.4 in light blue and 0–0.2 in dark blue).

Variants at the five previously unreported loci were visualised using LocusZoom (Fig. 2a–e) and forest plots for each of the lead variants (Supplementary Fig. S3) indicated low heterogeneity between studies for the lead variants (*I*^*2*^ = 0) at these loci in all cohorts. Stratified analyses for endometrioid and non-endometrioid cancers within the ECAC dataset for the five loci confirmed that overall effect estimates were similar to those from endometrioid endometrial cancer only analysis. We also ran analyses to compare effect estimate differences between the endometrioid and non-endometrioid stratified results, with no significant findings observed (Pdiff >0.05) (Supplementary Fig. S4).

We attempted to replicate associations for variants at the five loci by meta-analysis of three independent case-control studies (German cohort: 411 cases and 1122 controls, European Kazakh Meta Population (EMP): 307 cases and 84 controls, and Asian Kazakh Meta Population (AMP): 122 cases and 498 controls). The lead variant at the most significant locus, 12q21.2 (rs1677893), replicated in this independent German-Kazakh meta-analysis (OR = 1.21, 95%CI = 1.05–1.38, p = 0.006, Supplementary Table S5, visualised in forest plots in Supplementary Fig. S5). This was also supported by a Bayesian analysis of replication,^60^ with a calculated Generalised replication Bayes factor value of 0.71 (Supplementary Table S5). A significant association was also found for rs1382638 that is in strong linkage disequilibrium (LD) with rs1677893 (r^2^ = 0.99). Lead variants at two other loci (6q22.31 and 17q24.2) were not amenable to assay design (see Methods and Supplementary Table S5), but we tested closely linked variants for the two remaining loci (3p25.2 and 3q26.2). However, none of these were significantly associated with endometrial cancer risk in the independent German-Kazakh meta-analysis (Supplementary Table S5).

**Fig. 3:**
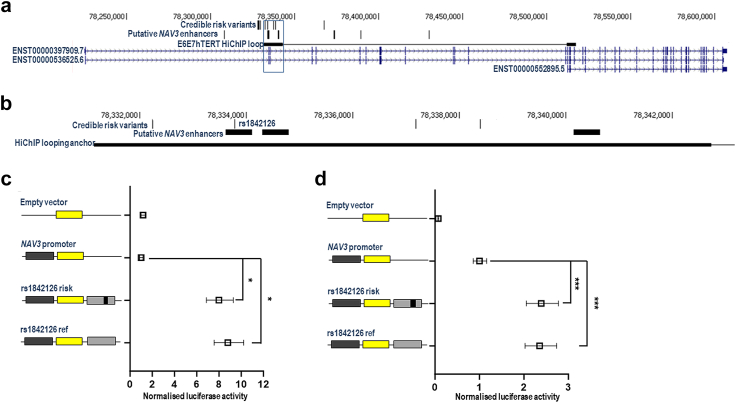
**Functional genetic analysis of the 12q21.2 risk locus.** (a) Mapping of credible risk variants, protein coding *NAV3* transcripts and promoter/enhancer HiChIP looping data at the 12q21.2 locus reveals colocalisation of risk variants with a HiChIP anchor that loops to the promoter region of a *NAV3* transcript (ENST00000552895.5). (b) Zooming into the highlighted box in Panel A, this panel illustrates that a risk variant (rs1842126) within the HiChIP anchor localises to a putative *NAV3* enhancer identified in endometrial tumours. Panels (c) and (d) show normalised luciferase activity from reporter gene assays involving the empty pGL3-Basic vector, a reporter gene construct containing the promoter of the ENST00000552895.5 *NAV3* isoform, and *NAV3* promoter reporter gene constructs with the putative enhancer carrying risk or reference alleles of rs1842126 in E6E7hTERT and Ishikawa cells, respectively. Error bars denote 95% confidence intervals for experiments conducted in triplicate. p values were determined by two-way ANOVA followed by Dunnett’s multiple comparisons test (∗p < 0.05, ∗∗∗p < 0.001).

We used available functional genomic data to identify gene targets of the risk variation at the new loci identified by the GWAS analysis. Firstly, we intersected credible risk variants (hereafter termed ‘risk variants’; Supplementary Table S2) with HiChIP promoter/enhancer chromatin looping data from normal and tumoural endometrial cell lines.^52^ Across the five loci, we identified 21 genes as candidate regulatory targets (Supplementary Table S10; exemplified by *NAV3* at the 12q21.2 locus in Fig. 3a). We next integrated expression quantitative trait locus (eQTL) data with risk variants to provide further evidence for candidate target genes. Using data from Genotype Expression Project (GTEx) tissues, we observed no robust associations (i.e., p < 1 × 10^−5^) in tissues considered relevant to endometrial cancer development (GTEx v8: subcutaneous adipose (n = 581), visceral omentum adipose (n = 469), ovary (n = 167), uterus (n = 129), and vagina (n = 141)). However, there was weak evidence of an association between expression of the candidate target gene *NAV3* in endometrial tissue and risk variants at 12q21.2 from an endometrium eQTL dataset^54^ (p = 2.2 × 10^−3^ for rs1842126; Supplementary Table S11). The credible risk variants at 12q21.2 were also found to be eQTLs for *NAV3* in adipose tissues in the TWINS UK cohort^61^ in the FIVEx eQTL browser^55^ (Supplementary Table S12).

To clarify the regulatory impact of risk variation on *NAV3*, we conducted reporter gene analysis. Risk variant selection for analysis involved mapping putative enhancers identified in endometrial tumours at the 12q21.2 locus.^62^ This mapping revealed one such enhancer that encompassed a risk variant (rs1842126) predicted to target *NAV3* (Fig. 3b). This putative enhancer also localised to a HiChIP looping anchor that interacted with the promoter of a *NAV3* transcript (ENST00000552895.5) in normal immortalised (E6E7hTERT) endometrial cells (Fig. 3a). Notably, this transcript is the most highly expressed *NAV3* isoform in nearly all GTEx tissues (https://gtexportal.org/home/gene/NAV3), including uterus, as well as in endometrial tumours from The Cancer Genome Atlas (TCGA) (data accessed using the GEPIA2 platform^63^). We cloned the putative enhancer containing rs1842126 into a reporter gene vector containing the corresponding *NAV3* promoter. Reporter gene assays in E6E7hTERT (Fig. 3c) and tumoural (Ishikawa) endometrial cells (Fig. 3d) confirmed an enhancer effect of the region containing the risk variant, with particularly strong effects in E6E7hTERT cells though no discernible allele-specific effects were seen in this system.

**Fig. 4:**
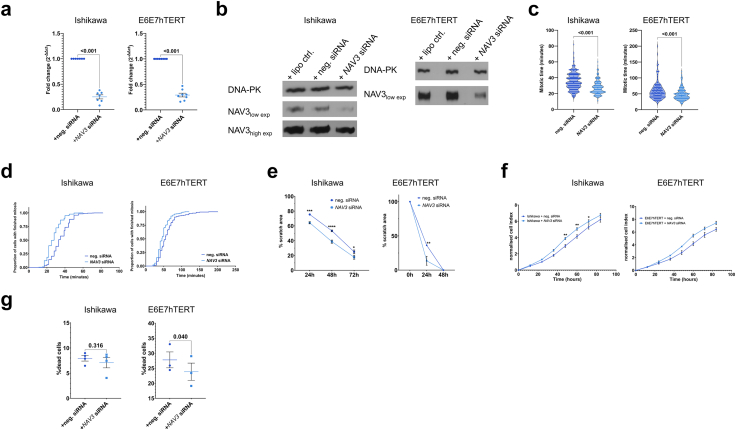
**Functional assessment after *NAV3* silencing in Ishikawa and E6E7hTERT cells.** (a) Fold changes after qRT-PCR for *NAV3* levels in Ishikawa cells (left panel), and E6E7hTERT cells (right panel). Each dot represents a biological replicate. p value indicated after paired t-test, (b) Western blot for NAV3 in Ishikawa cells (left panel), and E6E7hTERT cells (right panel) after silencing using siRNA, together with DNA-PK as housekeeper, high exp indicates higher exposure time, whereas low exp indicates lower exposure time, (c) Mitotic time per cell (in minutes) after silencing *NAV3* in Ishikawa cells (left panel), and E6E7hTERT cells (right panel). Each dot represents a cell in three biological experiments. p value indicated after unpaired t-test, (d) Proportion of cells that finished mitosis on the y-axis, time (in minutes) on the x-axis, after silencing *NAV3* in Ishikawa cells (left panel), and E6E7hTERT cells (right panel). Data shown from three biological experiments, (e) Percentage reduction of scratch area (relative to 0 h) on the y-axis and time (in hours) on the x-axis, after silencing *NAV3* in Ishikawa cells (left panel), and E6E7hTERT cells (right panel). Three biological experiments were performed in Ishikawa cells, and a single biological experiment was taken for E6E7hTERT cells. p value indicated with asterisk in multiple comparisons after ANOVA. ∗p < 0.05, ∗∗p < 0.01, ∗∗∗p < 0.001, ∗∗∗∗p < 0.0001, (f) Normalised cell index showing impedance in xCELLigence experiments after silencing *NAV3* in Ishikawa cells (left panel), and E6E7hTERT cells (right panel). Time in hours on the x-axis. Data shown from a single biological experiment with five technical replicates in both cell lines. p value indicated with asterisk in multiple comparisons after ANOVA. ∗p < 0.05, ∗∗p < 0.01, ∗∗∗p < 0.001, ∗∗∗∗p < 0.0001, (g) Percentage of dead cells after propidium iodide staining after silencing *NAV3* in Ishikawa cells (left panel), and E6E7hTERT cells (right panel). Treatment on the x-axis. Data shown from four biological experiments in Ishikawa and three biological replicates in E6E7hTERT cells. p value indicated after paired t-test.

TCGA data accessed through GEPIA2 showed *NAV3* expression is decreased by ∼59% in tumoural endometrium (n = 174) compared to paired normal samples (n = 339) (p_adj_ = 8.3 × 10^−64^). Given these findings, we performed functional follow-up studies to explore the role of *NAV3*, as its involvement in endometrial cancer has not been previously assessed. Firstly, expression of *NAV3* mRNA and NAV3 protein was confirmed in endometrial cell lines, including E6E7hTERT and Ishikawa (Supplementary Fig. S6). Both gene expression with qRT-PCR (Supplementary Fig. S6a) and protein analyses (Supplementary Fig. S6b) indicated strongly reduced *NAV3* expression in cancer cells compared with E6E7hTERT cells. We down-regulated *NAV3* expression using siRNAs in both cell lines with efficiencies of 70–80% (p < 0.001, Fig. 4a and b). Live-cell imaging indicated that *NAV3* downregulation resulted in a significant acceleration of mitotic progression (p < 0.0001 for both cell lines in three-way ANOVA with experiment as co-variable, Fig. 4c and d, Supplementary Fig. S7a and b). We also observed that *NAV3* downregulated cells migrated faster in scratch assays (Two-way ANOVA p values for silencing treatment p < 0.0001 for Ishikawa (F (1, 42) = 49.5) and p = 0.013 for E6E7hTERT (F (1, 6) = 12.24) cells, Fig. 4e, Supplementary Fig. S8) and exhibited increased impedance in xCELLigence profiling (Two-way ANOVA p value for silencing treatment = 0.023 for Ishikawa (F (1, 8) = 7.82) and p = 0.046 for E6E7hTERT cells (F (1, 5) = 6.88), Fig. 4f, biological replicate in Supplementary Fig. S9a) pointing to higher proliferation. Additionally, in the case of E6E7hTERT cells, *NAV3* silenced cells were more resistant to cell death (paired t-test p value = 0.04, Fig. 4g). Further endpoints tested did not show significant results (Supplementary Fig. S9b–d).

**Fig. 5:**
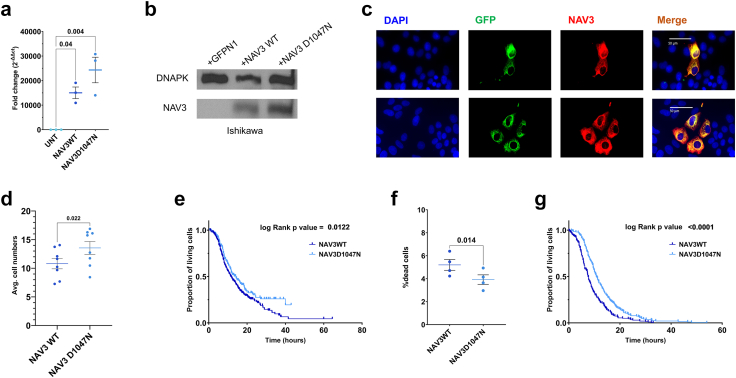
**Functional assessment after *NAV3* overexpression in Ishikawa cells.** (a) Fold changes after qRT-PCR for *NAV3* levels in Ishikawa cells transfected with pEGFPN1-NAV3WT and pEGFPN1-NAV3D1047N plasmids versus untreated controls. Each dot represents a biological replicate. p value indicated after paired t-test, (b) Immunoblotting of NAV3 and DNAPK in Ishikawa cells transfected with pEGFPN1, pEGFPN1-NAV3WT and pEGFPN1-NAV3D1047N, (c) Immunocytochemistry to visualize NAV3 overexpression in pEGFPN1-NAV3WT (upper panel) or pEGFPN1-NAV3D1047N (lower panel) transfected Ishikawa cells (NAV3 in red), with DAPI (for nuclear staining, in blue), and GFP fluorescence (in green), with the last panel showing the merged overlay, (d) Average cell numbers after transfecting Ishikawa cells with pEGFPN1-NAV3WT and pEGFPN1-NAV3D1047N plasmids at 72 h after transfection. Each dot represents a biological replicate. p value indicated after paired t-test, (e) Proportion of green cells remaining after transfecting Ishikawa cells with pEGFPN1-NAV3WT and pEGFPN1-NAV3D1047N plasmids. Each dot represents a cell from three biological experiments, with log rank p value shown, (f) Percentage of dead cells after DAPI staining after overexpression of pEGFPN1-NAV3WT and pEGFPN1-NAV3D1047N plasmids in Ishikawa cells. Data shown from four biological experiments. p value indicated after paired t-test, (g) Time to death in non-surviving green cells after transfecting Ishikawa cells with pEGFPN1-NAV3WT and pEGFPN1-NAV3D1047N plasmids. Each dot represents a cell from three biological experiments, with log rank p value shown.

We also investigated the effect of *NAV3* overexpression. NAV3 has been previously described as a regulator of breast cancer progression and a dominant-negative mutation D1047N has been described.^57^ To further establish the role of *NAV3* in endometrial cancer, we overexpressed EGFP-fused wildtype (EGFPN1-NAV3WT) and mutant (EGFPN1-NAV3D1047N) *NAV3* in Ishikawa cells, with the latter being used as functionally inactive negative control. We obtained high levels of *NAV3* overexpression in Ishikawa cells as confirmed via qRT-PCR (p (t-test) = 0.04 and 0.004 for EGFPN1-NAV3WT or EGFPN1-NAV3D1047N cells compared to untreated cells, Fig. 5a), western blotting (Fig. 5b) and immunocytochemistry (Fig. 5c). Despite the high overall NAV3 expression, we note that only a subfraction of cells were overexpressing the large plasmid, while transfection rates were higher for the EGFPN1-only construct (likely due to the smaller plasmid size). Therefore, we monitored only successfully transfected GFP-labelled cells in time-lapse microscopy: NAV3 wildtype overexpressing Ishikawa cells showed a significant reduction in cell number compared to cells overexpressing mutant NAV3 (p (t-test) = 0.02, Fig. 5d, Supplementary Fig. S7c) and decreased survival of cells over-expressing wildtype NAV3 compared to the functionally inactive D1047N mutant (p (t-test) = 0.012, Fig. 5e). There was a higher fraction of dead cells 24 h after overexpressing wildtype NAV3 as compared to the functionally inactive D1047N mutant (p (t-test) = 0.014, Fig. 5f) as well as significantly accelerated cell death in the fraction of dying cells (Fig. 5g). Taken together, NAV3 downregulation accelerated tumour cell division and migration of endometrial cells, whereas its overexpression accelerated tumour cell death.

## Discussion

In the present study, we performed the largest GWAS meta-analysis for endometrial cancer to date, combining data from the Endometrial Cancer Association Consortium with genotyping data from four biobanks: UK Biobank, FinnGen, Estonian Biobank and Biobank Japan. We identified five previously unreported genomic regions associated with endometrial cancer, and two further genomic regions from gene-based analysis. We also identified an independent signal at a known locus (8q24.21), confirmed fourteen previously reported independent susceptibility signals at GWS,^21^, ^22^, ^23^ and report on twenty further sub-genome-wide loci at p < 10^−6^. BFDPs indicated that all GWS loci are likely to be true associations (BFDPs < 4%).

For three of the five previously unreported loci, our gene-based MAGMA analyses also uncovered underlying candidate genes at genome-wide significance: *PPARG* at 3p25.2, *NAV3* at 12q21.2 and *BPTF* at 17q24.2. *PPARG* encodes Peroxisome Proliferator-Activated Receptor gamma (PPAR-γ), a transcription factor regulating glucose metabolism and epithelial differentiation of trophoblast tissue. PPAR-γ is involved with insulin sensitivity, adipokine and oestrogen signalling, that are pathways relevant for endometrial cancer. PPAR-γ has been reported to regulate the expression of genes involved in the DNA damage response in an inflamed endometrium,^64^ and PPARγ ligand telmisartan has been suggested as a treatment option for endometrial cancer.^65^ *NAV3* encodes Neuron Navigator 3, a modulator of cell migration. NAV3 has been proposed as a candidate tumour suppressor protein in breast cancer, colon cancer and uterine leiomyoma, though a role for endometrial cancer had not been described.^57^^,^^66^^,^^67^ *BPTF* encodes the Bromodomain PHD Finger Transcription Factor, the largest subunit of the nuclear remodelling factor complex. Trimethylation of histone H3 lysine 4 (H3K4me3), a mark for actively transcribed promoters, is recognised by the PhD domain of BPTF within this complex.^68^ BPTF is a target and regulator of MYC and exerts pro-tumourigenic functions in diverse cancers including melanoma,^69^ hepatocellular carcinoma,^70^ or ovarian cancer.^71^ On the other hand, BPTF targeting with the bromodomain inhibitor bromosporine has been reported to exert anti-tumour effects against triple-negative breast cancer cells.^72^ Genes in vicinity of risk variants at the two other genome-wide significant loci, 3q26.2 and 6q22.31, include *MECOM* whose mutation is known as a somatic driver of endometrial cancer,^73^ *HSF2* encoding an oestrogen-regulated heat-shock factor,^74^ and *GJA1* encoding the gap junction protein connexin-43 which may regulate uterine decidualisation and whose expression decreases with increased grade of EC.^75^^,^^76^

We functionally followed the signal at 12q21.2 that was the strongest identified risk locus in our GWAS meta-analysis after it had been previously sub-genome-wide significant.^22^ The lead SNP representing the risk at this locus, arising from our meta-analysis in European and East-Asian populations, replicated in the genotyped German (European) and Kazakh (European and Asian) populations, indicating that this is likely a universal susceptibility locus. Functional genomic data from endometrial tumours and cell lines revealed *NAV3* as a candidate regulatory target of risk variation at this risk locus. HiChIP looping data supported an interaction between the risk region and the *NAV3* promoter whereas eQTL data were inconclusive and not supported by reporter gene assays. It is possible that activation of the *NAV3* promoter is not captured by our assays or by the single variants investigated. Alternatively, the risk variants could act on other genes than *NAV3*, or in other tissues. To clarify the potential role of NAV3 in endometrial cells, we performed a more extensive functional investigation. High protein expression in a non-malignant endometrial cell line and the markedly reduced expression in all tested endometrial cancer lines further indicated a potential role of NAV3 as a tumour suppressor in the endometrium and was consistent with TCGA expression data. In silencing and overexpression experiments, we found convergent evidence for accelerated mitosis and growth in NAV3 downregulated cells but accelerated cell death in NAV3 overexpressing cells. Previous studies have reported an association of copy number changes in *NAV3* with epithelial cell cancer pathogenesis,^77^ colorectal cancers and adenomas^66^^,^^78^ while suggesting that this effect may be mediated through inflammatory pathways. Another study showed that silencing *NAV3* in breast cancer cells increased cell migration and metastasis,^57^ which is concordant with our own findings of a possible increase in migratory capacity in endometrial cells upon *NAV3* silencing. Thus despite incomplete evidence for a direct regulatory mechanism, our findings, together with prior evidence, support the hypothesis that risk variation at this locus increases cancer susceptibility by downregulating *NAV3* expression.

While we functionally followed the top signal of our GWAS, a present limitation of our study is the lack of further information on the other four regions which warrant further functional investigation. It will also be interesting to see whether the candidate causal variants at the five loci can be refined further in fine-mapping approaches as other population data become available. A strength of our study is the large sample size that was achievable by combining all previous GWAS analyses. We consider our study representative because none of the biobanks or recruiting hospitals involved had specifically selected for family history of cancer or early age at diagnosis. Future studies including additional and multi-ethnic case-control cohorts will improve power and enable the detection of associated genomic risk loci. The generation of well-powered relevant eQTL datasets may enable colocalization analyses and fine-mapping to identify causal variants and genes.

In summary, our GWAS meta-analysis increased the number of known genetic risk loci for endometrial cancer by one-third and identified potential candidate genes at these risk loci. Assuming a log–additive association with risk, these new loci are estimated to account for an additional 1.2% of the 2-fold familial relative risk of endometrial cancer. We further provide functional evidence for NAV3 to be a tumour suppressor in endometrial cells. Larger case-control studies in the near future will likely uncover further genetic risk variants that can explain more of the genetic heritability of this complex disease, ultimately leading to improved polygenic risk scores and treatment options.

## Contributors

Bioinformatic analysis—D.R., X.W., T.L., N.I., P.F.K., D.M.C., D.M.G., T.A.O. Wet-lab experiments—D.R., Y.Z., D.M.G., B.N., C.J.C., P.S., B.W., P.H., F.H. and K.B. Experimental data analysis—D.R., D.M.G., and T.D. Clinical samples and data curation—N.H., N.T., N.D., T.Y., Z.B.Y., A.V.K., P.Hi, F.A., D.A., M.W.B., C.B., D.D.B., C.C., M.A.C., L.S.C., I.DV., W.D., M.D., D.F.E., J.E., P.A.F., C.M.F., G.Ga., G.G.G., E.L.G., H.R.H., D.J.H., D.L.K., P.K., J.V.L., D.L., L.L., G.L.M., J.N., K.O., A.V.P., P.D.P.P., T.R.R., F.R., H.A.R., M.R., C.S., R.J.S., VW.S., X-O.S., M.C.S., E.T., I.T., C.T., N.W., H.Y., W.X., W.Z. Resources—R.M., Y.Y. Manuscript writing and editing—D.R., D.M.G., T.D., T.A.O. Conceptualization—D.R., T.D., T.A.O. All authors read and agreed to this version of the manuscript.

## Data sharing statement

GWAS meta-analysis summary statistics are available at GWAS Catalog (GCP001067, GCST90454186). Experimental data are provided in data file “Source data.xlsx”. Full western blots are provided in a PDF file of the same name.

## Declaration of interests

D. Ramachandran received intramural funding from Hannover Medical School. M. Clark. has stock options in AbbVie and is employed by AbbVie on work not related to current manuscript. 1U01CA250476-01A1 to I. De Vivo and G. L. Mutter. NCI P30CA008748, U01CA250476 to M. Du. J. Emons receives honoraria for lectures from Pfizer, Eisai, AstraZeneca, Novartis, participates in the advisory board for these and MSD, receives travel money from AstraZeneca. Grant awards 3U01-CA199277-07S1 and 3U01-CA199277-08S1 from the NCI to J. V. Lacey for whole genome sequencing for participants in the California Teachers Study, which contributed data to this manuscript. P. A. Fasching receives grants from BioNTech, Cepheid, Pfizer; consulting fees, honoraria for lectures, and participates in an advisory board for Novartis, Pfizer, Roche, Daiichi-Sankyo, AstraZeneca, Lilly, Eisai, Merck Sharp & Dohme, Pierre Fabre, SeaGen, Agendia, Sanofi Aventis, Gilead, Mylan. D. L. Kolin holds stocks of Abbott laboratories, Alcon Inc., Becton Dickinson, Novartis, Pfizer, and UnitedHealth Group. G. L. Mutter received consulting fees from Bayer as personal consultant in pathology to perform diagnostic safety reads for clinical trials. A. V. Patel is on the NCI board of scientific counsellors. NIH funding to H. Risch. E. Tham received grants from Region Stockholm, the Swedish Childhood Cancer Fund (Barncancerfonden) and the Swedish Cancer Fund (Cancerfonden). E. Tham is a board member of Anna Dahlbäck’s memorial fund. Grants from CRUK and Genome Canada to D. Easton. NIH grant to P. Kraft. CRUK grants to P. Pharoah. D. Lambrechts receives annual funding from VIB. D. Glubb received payment from the University of Sharjah for grant reviewing. Grant from Wilhelm Sander Foundation to T. Dörk and P. Hillemanns. T. A. O’Mara received funding from the US Department of Defence and Worldwide Cancer Research. None of the sponsors had any role in the design, data generation or result interpretation in this study. All other authors declare no competing interests.

## Appendix ASupplementary data

Supplementary Table S1 Supplementary Table 1: Studies in the meta-analysis.

Supplementary Table S2 Supplementary Table 2: Set of credible risk variants at each of the five loci.

Supplementary Table S3 Supplementary Table 3: Patient demographics for the wet-lab genotyping cohorts.

Supplementary Table S4 Supplementary Table 4: Fluidigm SNPs genotyping design with call rates, HWE information and number of samples genotyped per SNP.

Supplementary Table S5 Supplementary Table 5: Results from meta-analysis and logistic regression analysis after wet-lab validation genotyping in German and Kazakh cohorts for the five GWS loci.

Supplementary Table S6 Supplementary Table 6: siRNA, qPCR primer sequences used in the study.

Supplementary Table S7 Supplementary Table 7: Summary statistics generated after meta-analysis for sub-GWS candidate loci identified in this study at p < 10E-6.

Supplementary Table S8 Supplementary Table 8: Results from COJO analysis.

Supplementary Table S9 Supplementary Table 9: MAGMA Summary statistics generated after meta-analysis with window of 20kb.

Supplementary Table S10 Supplementary Table 10: Candidate gene targets arising from HiChip data.

Supplementary Table S11 Supplementary Table 11: eQTLs at 12q21.2 in endometrium dataset from Mortlock et al.^54^

Supplementary Table S12 Supplementary Table 12: eQTLs at 12q21.2 in adipose from FIVEx eQTL browser.

Full Western Blots

Supplementary Figs. S1–S9 Supplementary Fig. 1: Cluster plots for the genotyped SNPs at the five loci. Supplementary Fig. 2: Plasmid maps for EGFPN1, EGFPN1-NAV3-WT, EGFN1-NAV3-D1047N. Supplementary Fig. 3: forest plots for all genome-wide significant signals in this study. Supplementary Fig. 4: forest plots and effect estimate for all genome-wide significant signals in this study, in the overall ECAC dataset, and in endometrioid and non-endometrioid cancers from the ECAC dataset. Supplementary Fig. 5: forest plots for variants at chr 12 (NAV3) after wet-lab genotyping. Supplementary Fig. 6: NAV3 expression in different EC cell lines, by (a) real time PCR and (b) western blotting. Shown are endometrial cancer cell lines ANC3A, JHUEM-14, MFE-296 and Ishikawa, as well as the non-cancerous immortalized E6E7hTERT. High exp indicates higher exposure time, whereas low exp indicates lower exposure time. Supplementary Fig. 7: (a-b) Representative images of time lapse microscopy to analyse mitotic time duration in Ishikawa and E6E7hTERT cells after silencing treatment, and (c) of green labelled cells analysed in Ishikawa cells after NAV3 overexpression. Supplementary Fig. 8: Representative images from scratch analysis in (a) Ishikawa and (b) E6E7hTERT after silencing *NAV3*. Supplementary Fig. 9: (a) xCELLigence impedance profile in Ishikawa cells and E6E7hTERT cells with neg. control siRNA and after silencing *NAV3*, N = 1 biological experiment, with 4 technical replicates. p value indicated after overall ANOVA across all time points. (b) Results from flow cytometry-based cell cycle analysis in Ishikawa cells. Values shown are % of all cells, n = 3 biological experiments, p value after unpaired t-test. (c) ATP levels measured after *NAV3* silencing in Ishikawa and E6E7hTERT cells after 72H. p value indicated after paired t test. N = 4 and 3 biological experiments (respectively) with 3 technical triplicates each. (d) ATP levels measured after *NAV3* overexpression in Ishikawa cells after 24H. p value indicated after paired t test. N = 2 biological experiments with 3 technical triplicates. (e) Biological replicate of Fig. 5b, showing immunoblotting of NAV3 and DNAPK in Ishikawa cells transfected with pEGFPN1, pEGFPN1-NAV3WT and pEGFPN1-NAV3D1047N.
