# Supplementary material for: GWAS meta-analysis identifies five susceptibility loci for endometrial cancer
Source: eBioMedicine. 2025 Jul 8;118:105830. doi: 10.1016/j.ebiom.2025.105830 (PMC12275056; doi:10.1016/j.ebiom.2025.105830)

Fig4B WB
Ishikawa

DNA-PK

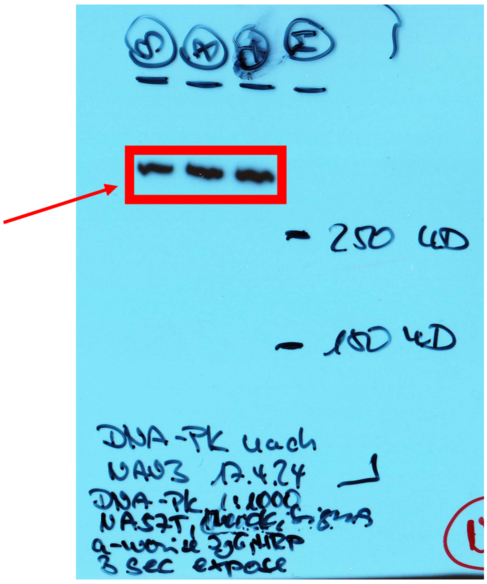

EM-E6E7hTERT

DNA-PK

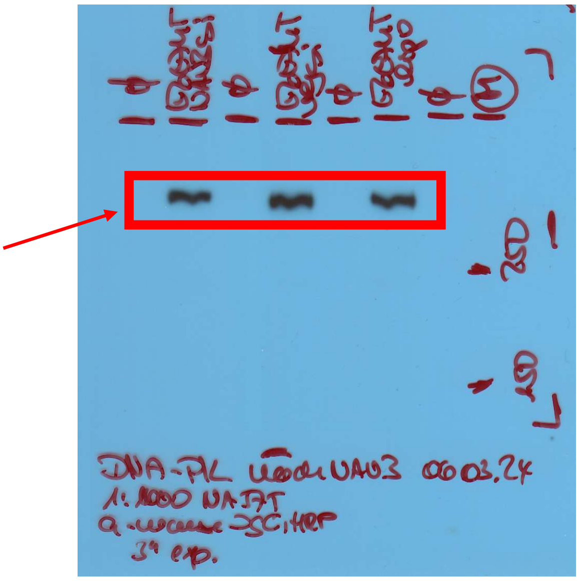

NAV3high exp

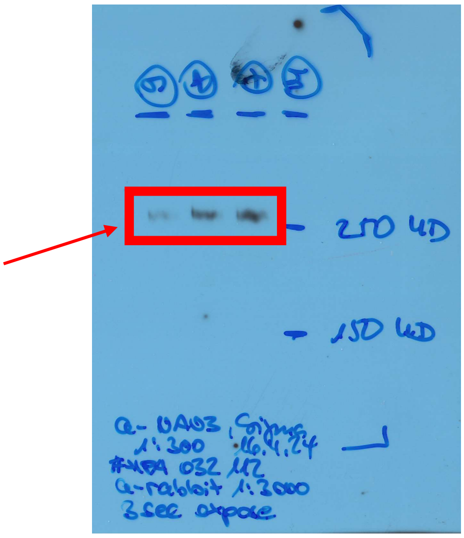

NAV3 low exp

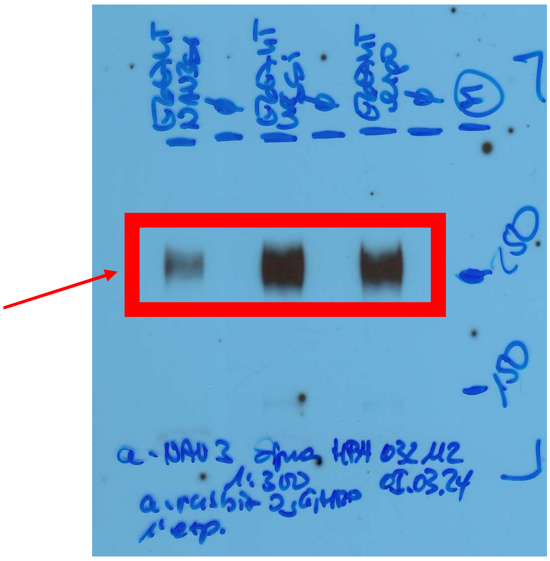

NAV3low exp

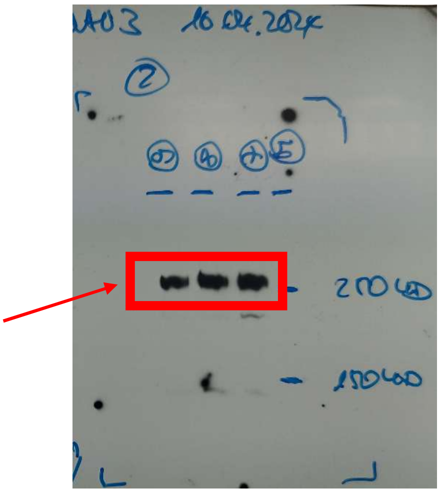

Fig5B WB

Ishikawa

DNA-PK

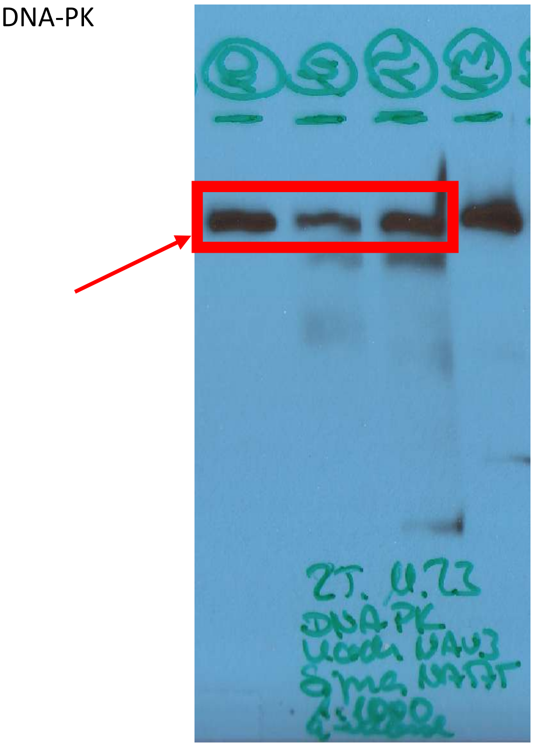

NAV3high exp

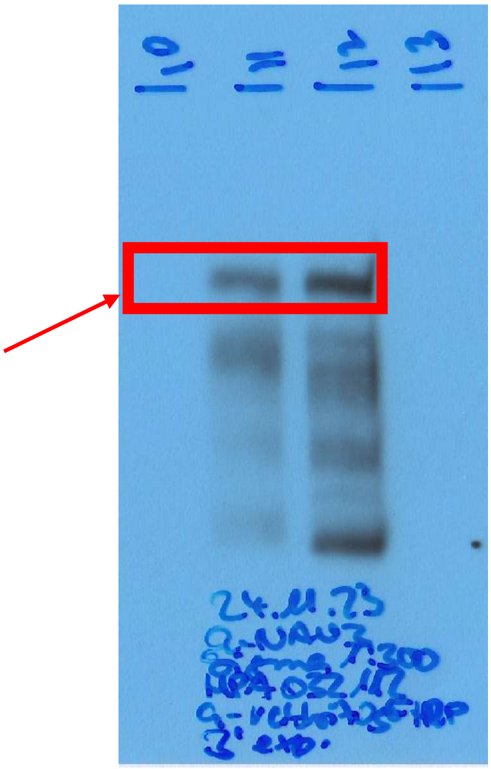

SFig3B WB
Ishikawa

DNA-PK

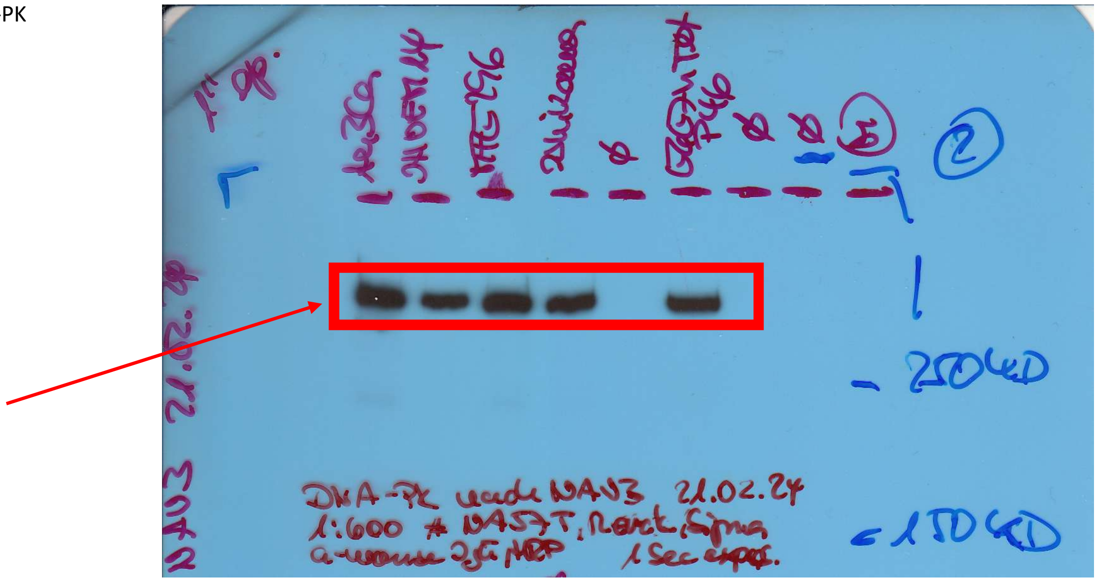

NAV3 low exposure

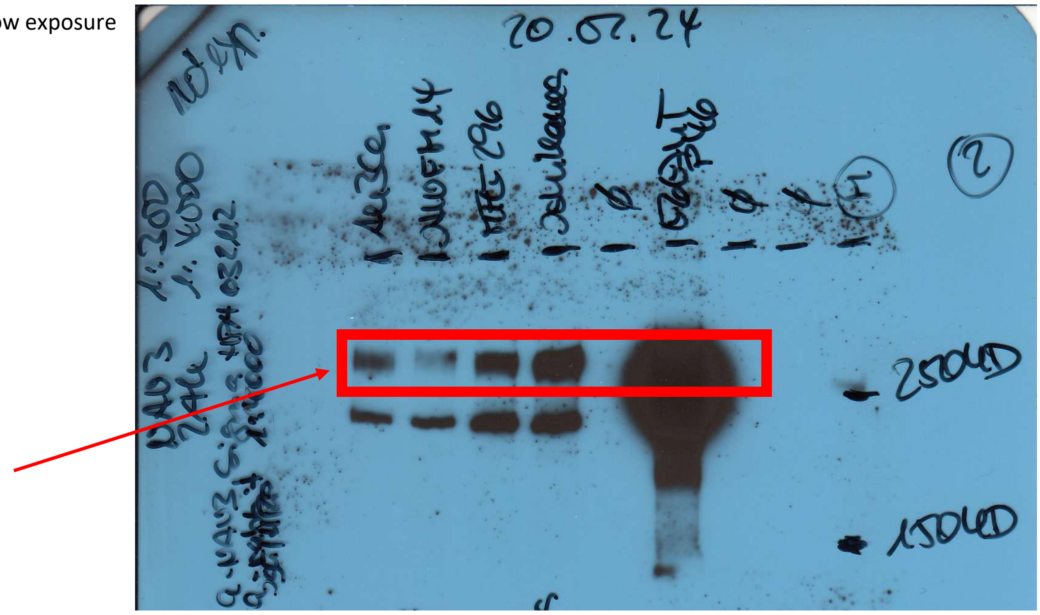

NAV3 high exposure

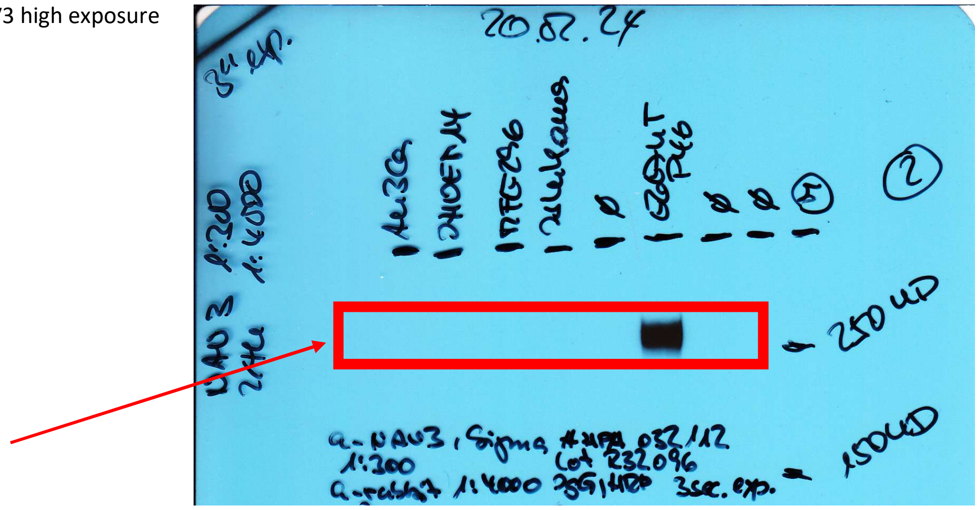

SFig9E WB

Ishikawa

DNA-PK

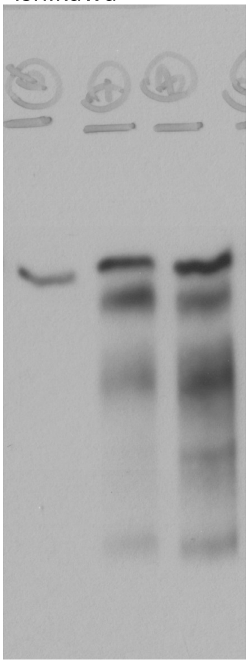

NAV3high exp

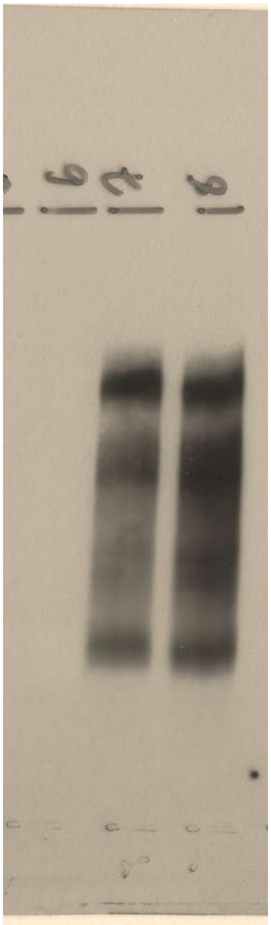

Supplement: Full Western Blots [file mmc13.pdf]
