# Supplementary material for: GWAS meta-analysis identifies five susceptibility loci for endometrial cancer
Source: eBioMedicine. 2025 Jul 8;118:105830. doi: 10.1016/j.ebiom.2025.105830 (PMC12275056; doi:10.1016/j.ebiom.2025.105830)

Supplementary Figure 1

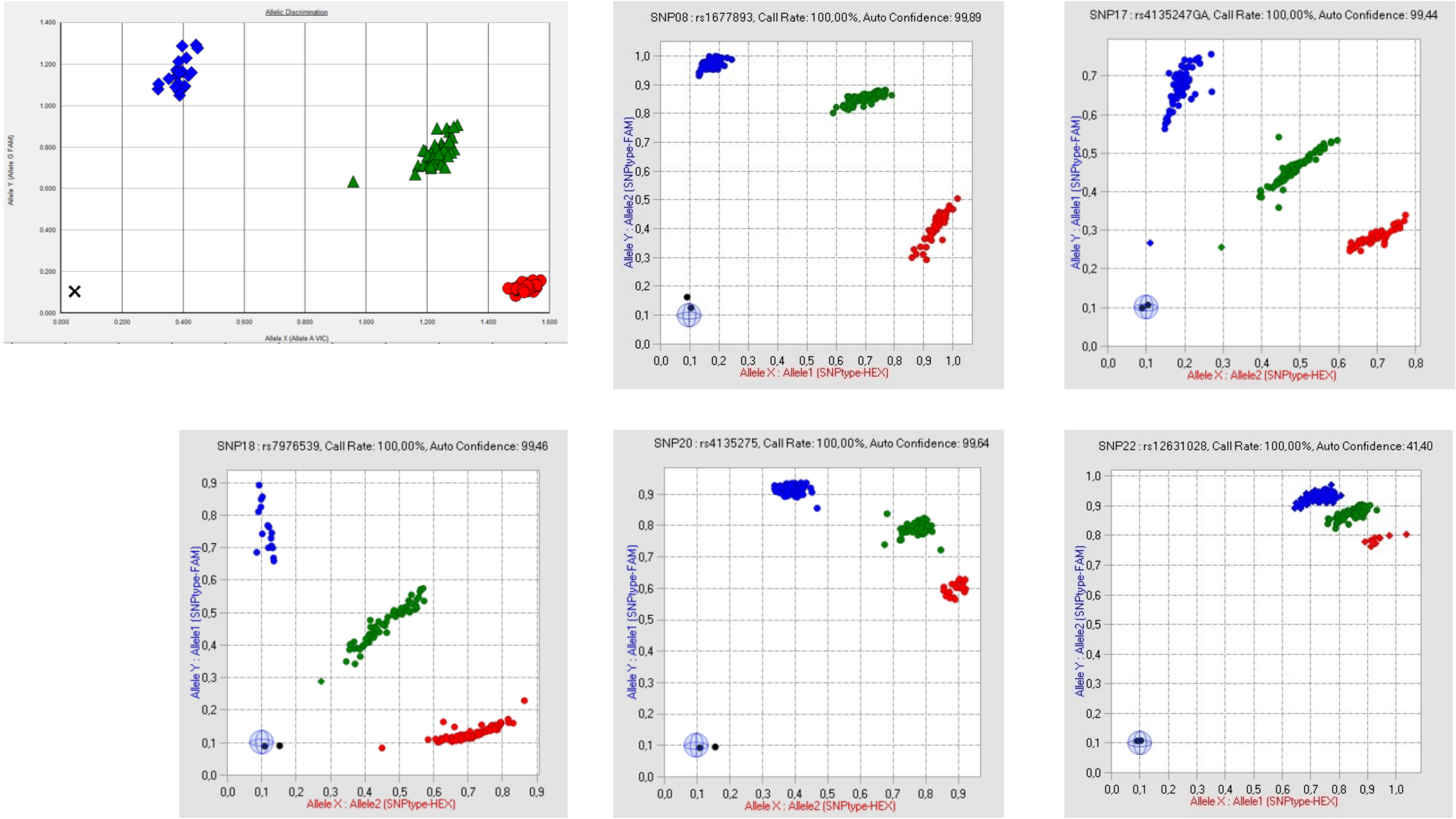

Supplementary Figure 2

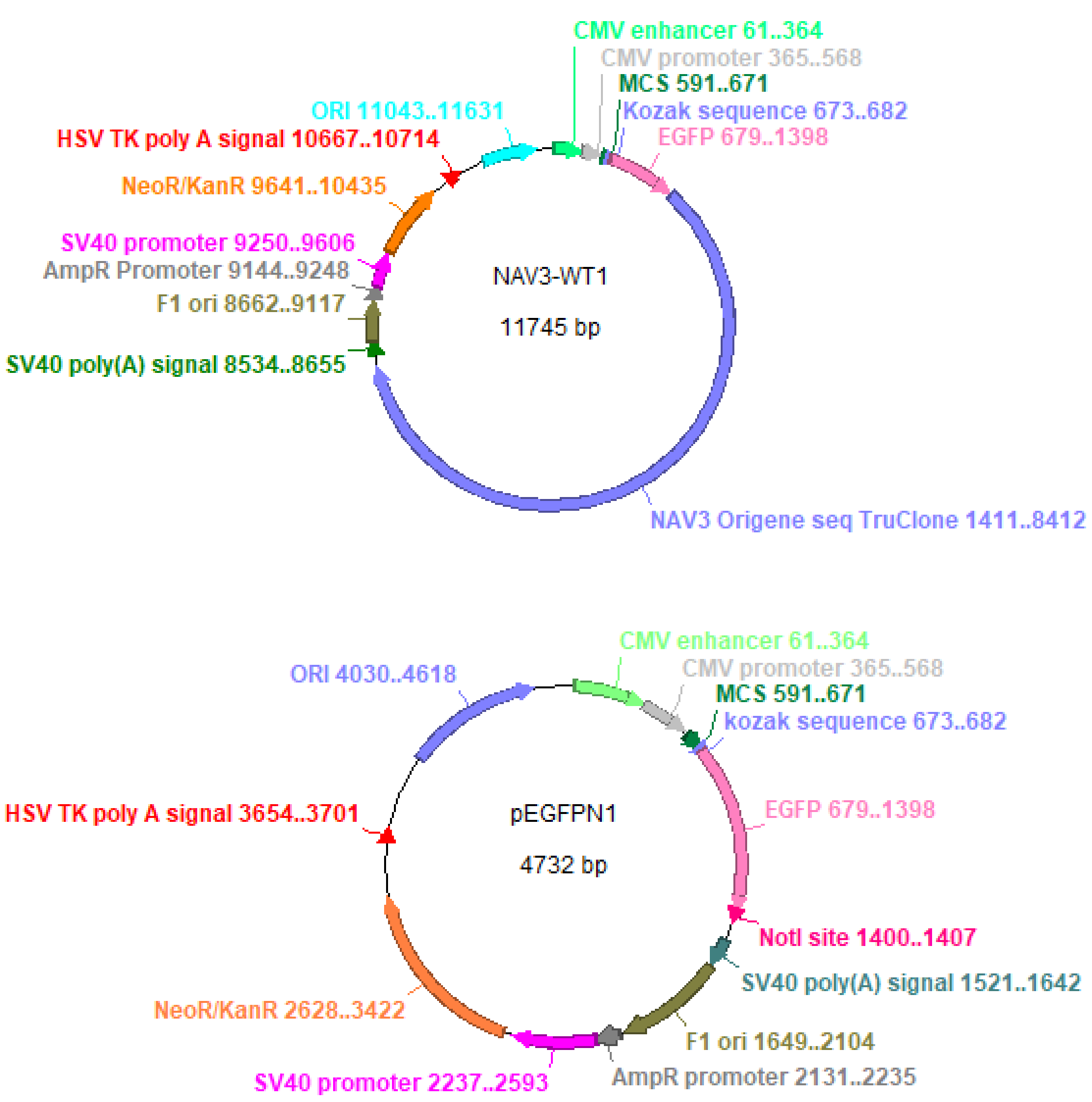

Supplementary Figure 3

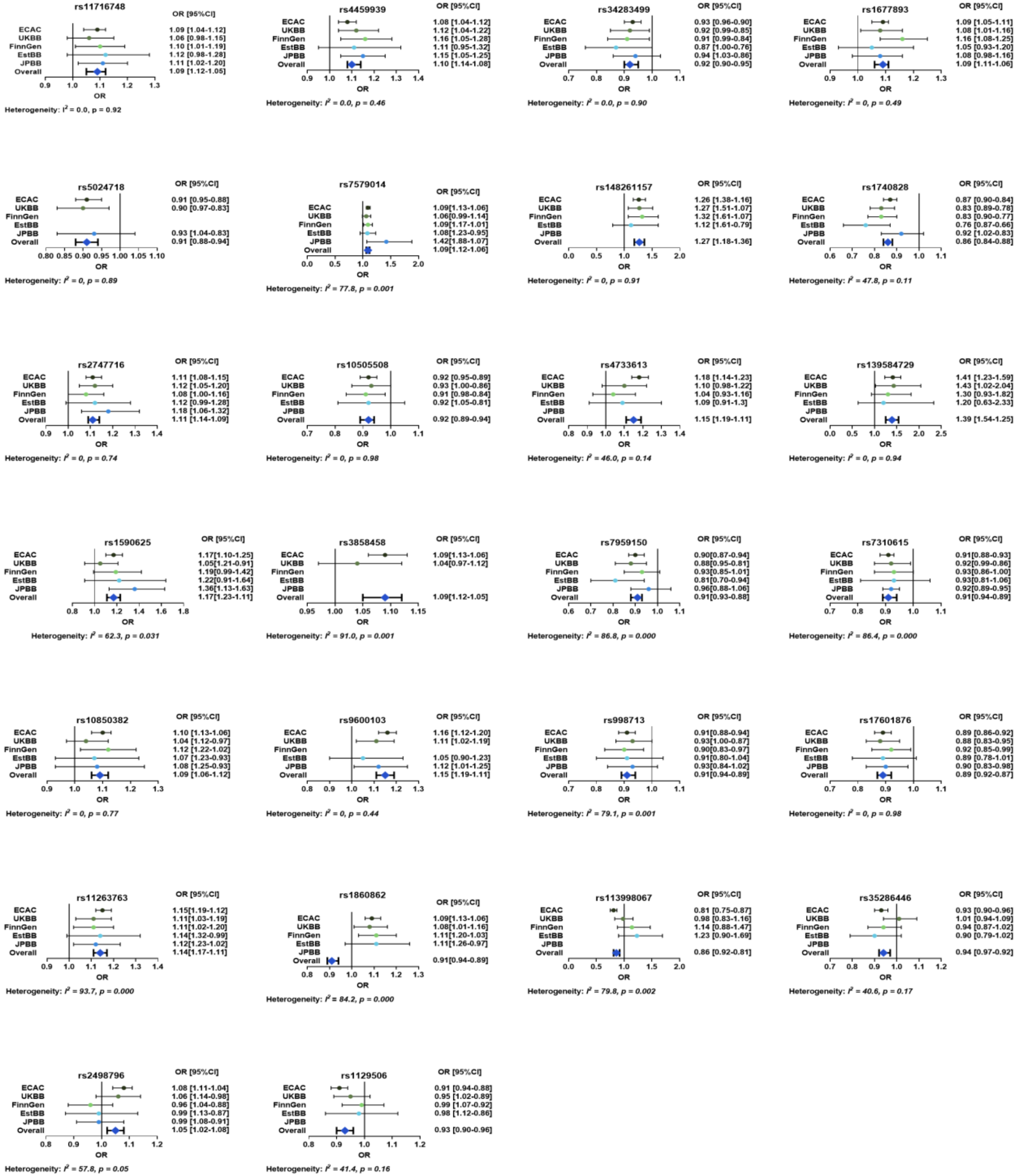

Supplementary Figure 4

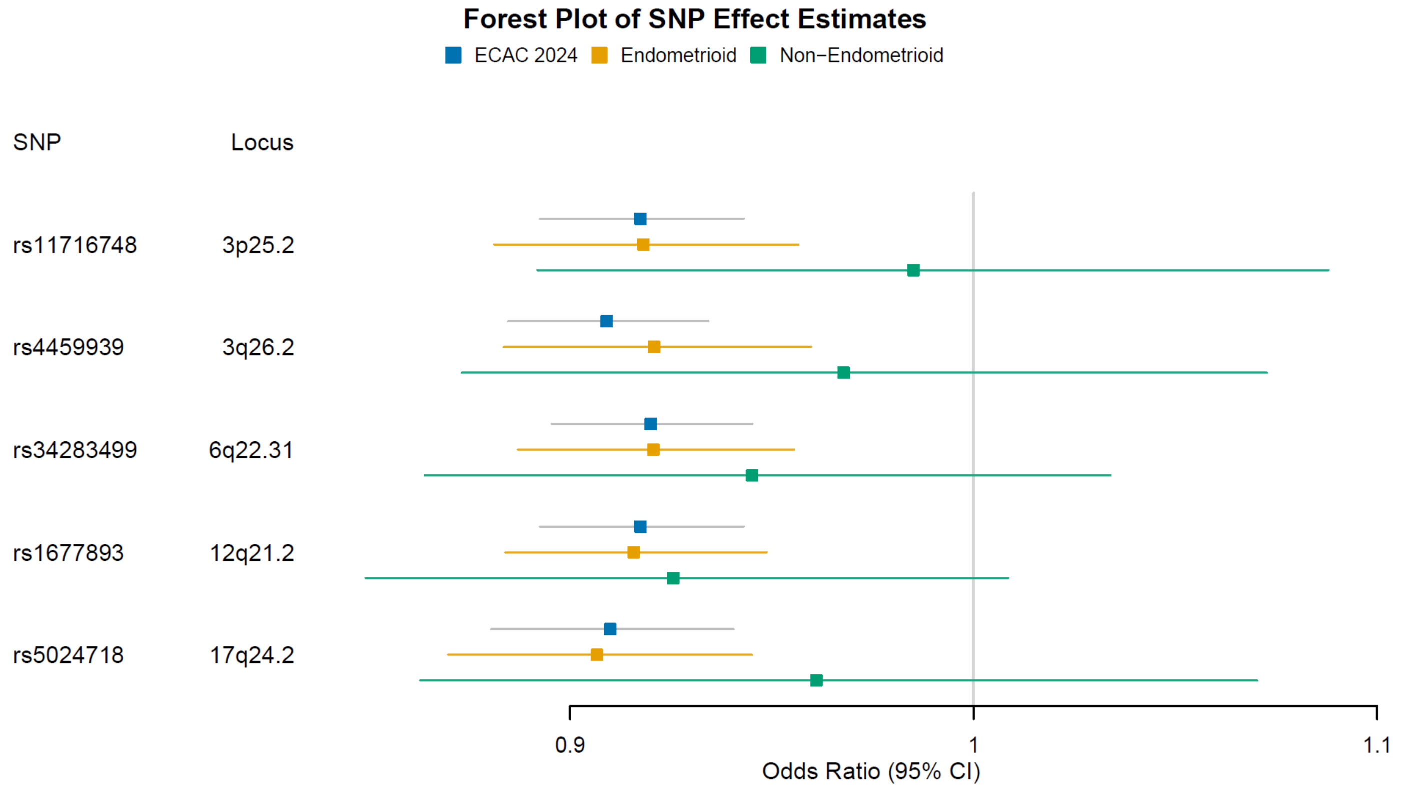

Locus	MarkerName	chr:pos_b37	EA	OA	OR (95%CI)			EEC v NEEC
					Present study	2018 study EEC only	2018 study NEEC only	P_{diff}
3p25.2	rs11716748	3:12336399	G	A	0.92 (0.89 - 0.94)	0.92 (0.88 - 0.96)	0.99 (0.89 - 1.09)	0.79
3q26.2	rs4459939	3:168643983	C	T	0.91 (0.88 - 0.93)	0.92 (0.88 - 0.96)	0.97 (0.87 - 1.07)	0.85
6q22.31	rs34283499	6:122402574	T	C	0.92 (0.9 - 0.95)	0.92 (0.89 - 0.96)	0.95 (0.86 - 1.03)	0.92
12q21.2	rs1677893	12:78338386	T	A	0.92 (0.89 - 0.94)	0.92 (0.88 - 0.95)	0.93 (0.85 - 1.01)	0.97
17q24.2	rs5024718	17:65892587	T	C	0.91 (0.88 - 0.94)	0.91 (0.87 - 0.95)	0.96 (0.86 - 1.07)	0.83

Supplementary Figure 5

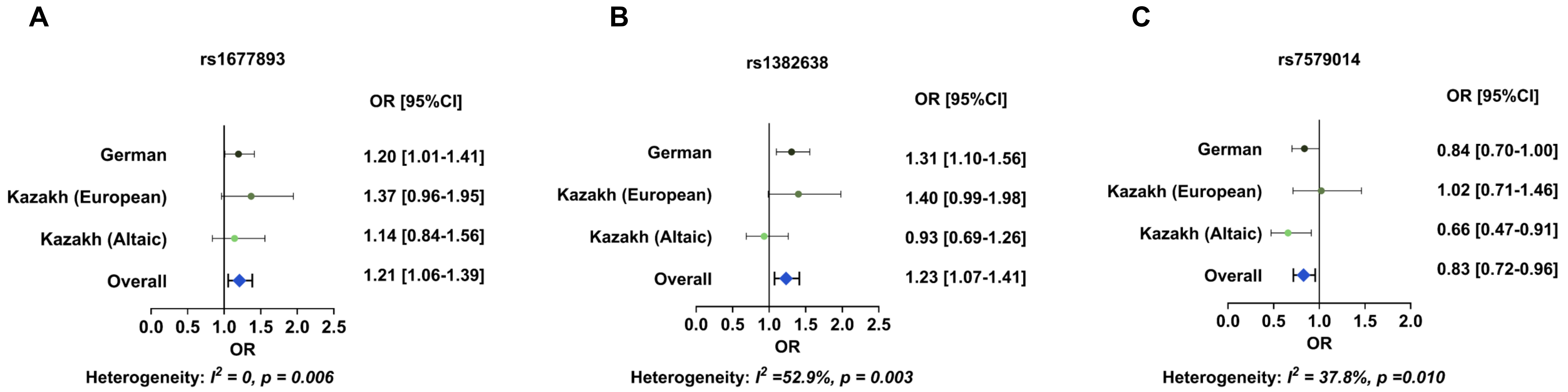

Supplementary Figure 6

A

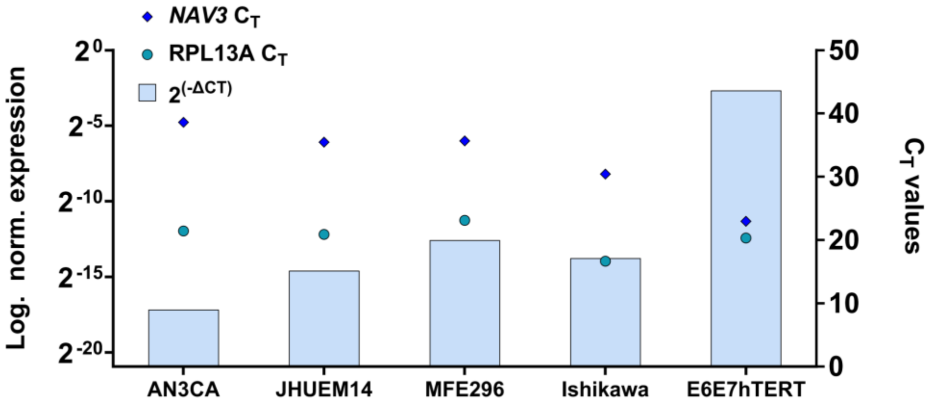

B

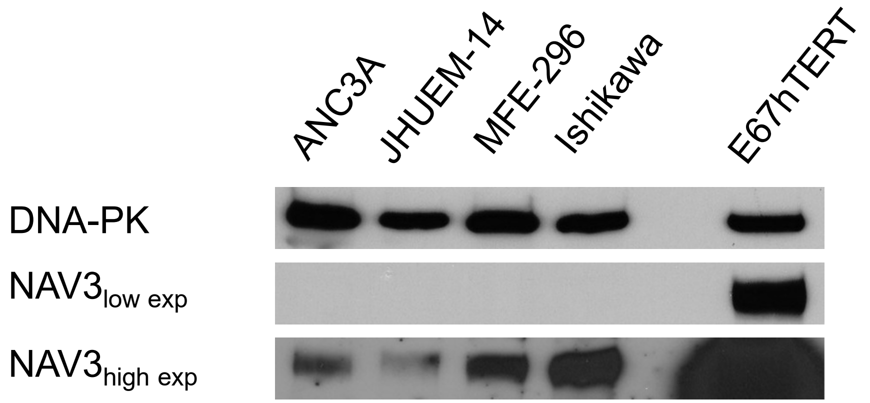

Supplementary Figure 7

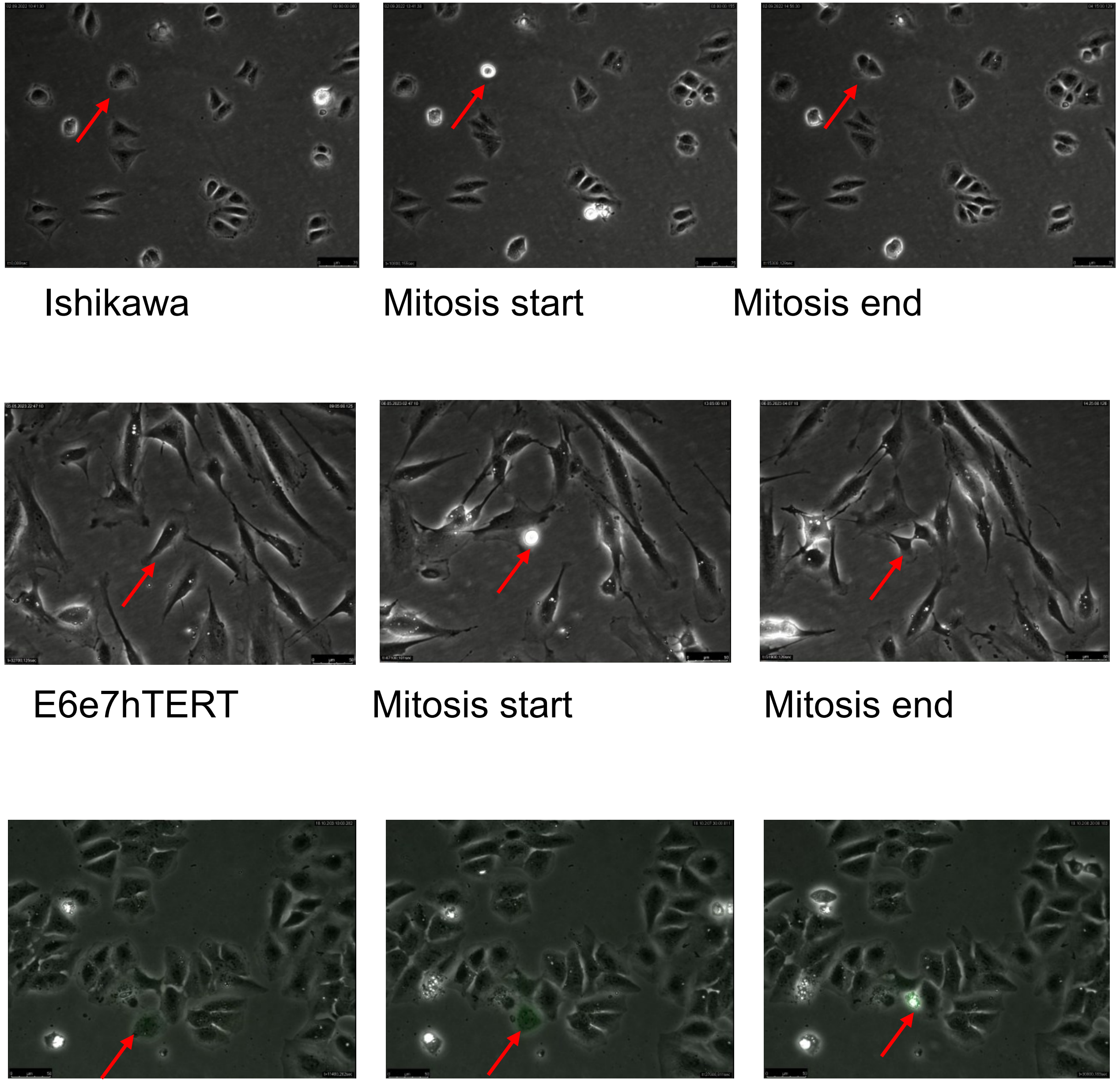

A

Ishikawa — Mitosis start — Mitosis end

B

E6e7hTERT — Mitosis start — Mitosis end

C

Start (GFP-NAV3) — Death

Supplementary Figure 8A

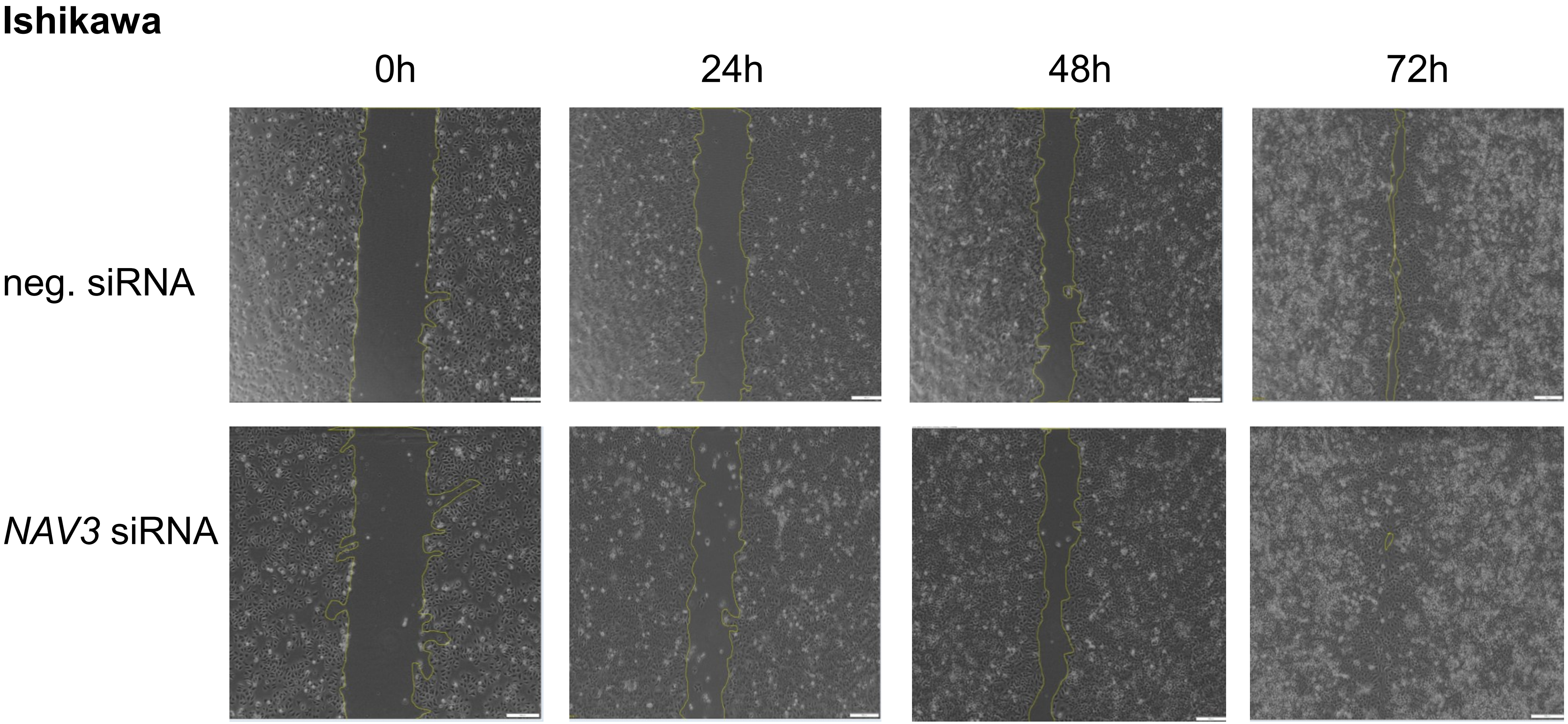

Supplementary Figure 8B

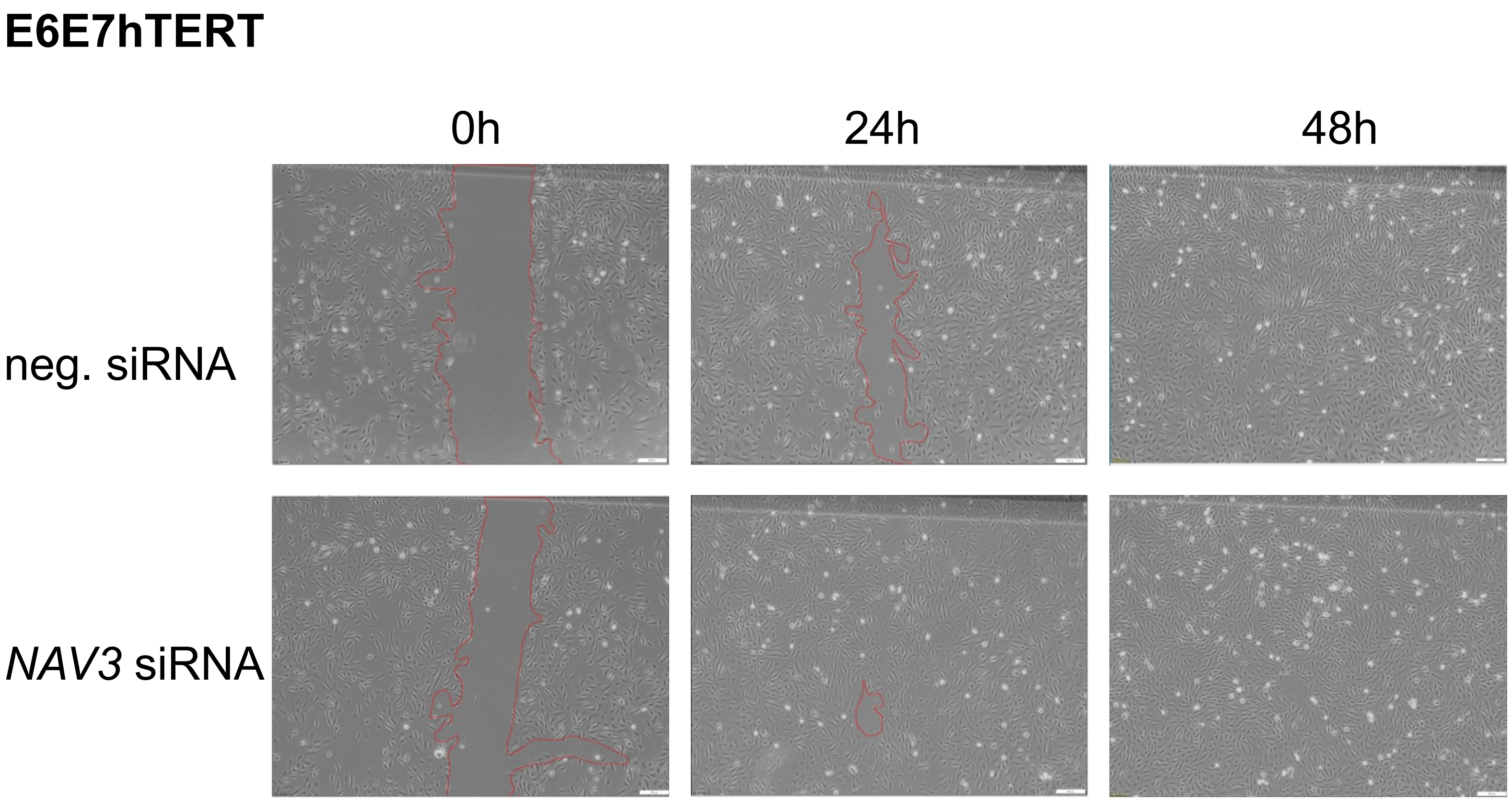

Supplementary Figure 9

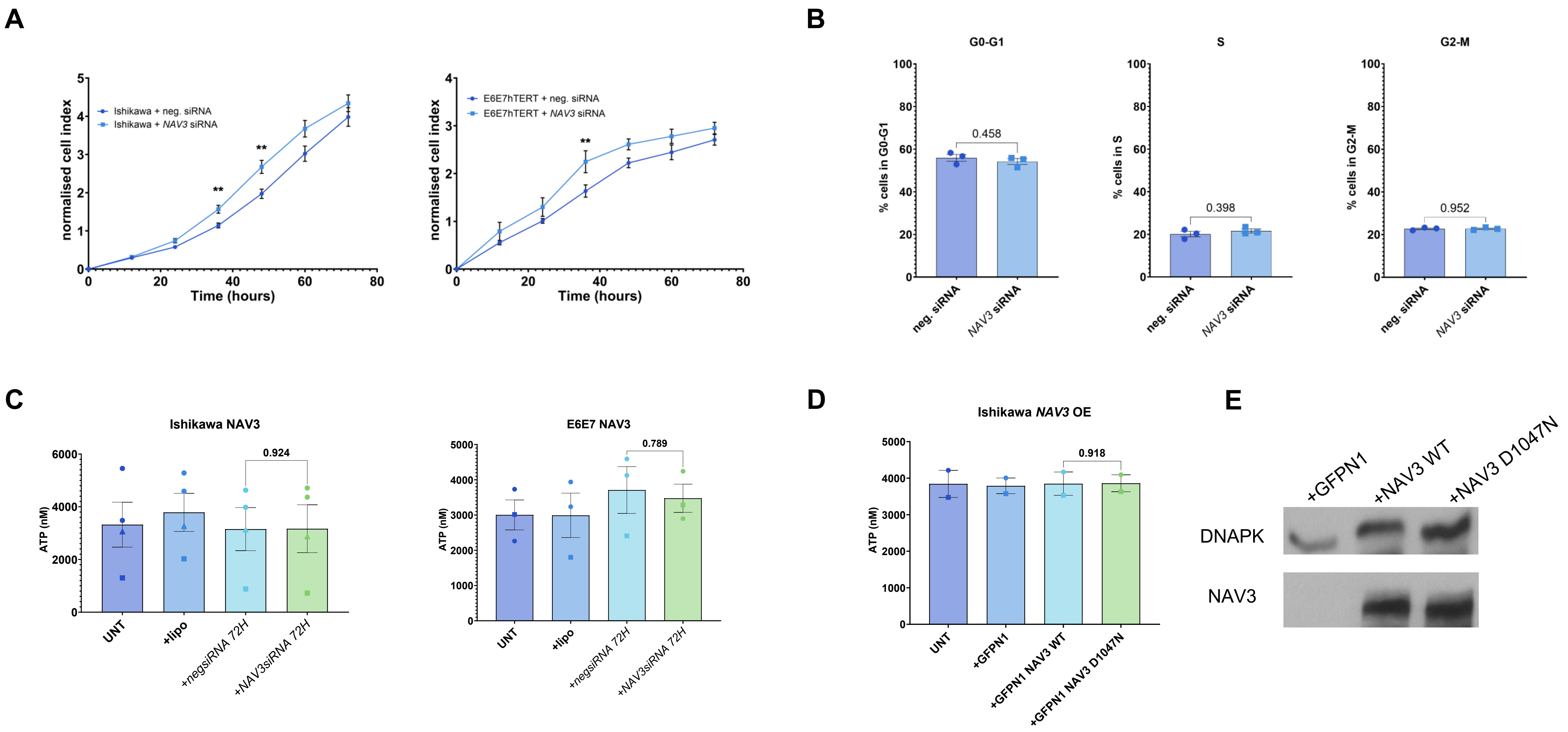

Supplement: Supplementary Figs. S1–S9 — Supplementary Fig. 1: Cluster plots for the genotyped SNPs at the five loci. Supplementary Fig. 2: Plasmid maps for EGFPN1, EGFPN1-NAV3-WT, EGFN1-NAV3-D1047N. Supplementary Fig. 3: forest plots for all genome-wide significant signals in this study. Supplementary Fig. 4: forest plots and effect estimate for all genome-wide significant signals in this study, in the overall ECAC dataset, and in endometrioid and non-endometrioid cancers from the ECAC dataset. Supplementary Fig. 5: forest plots for variants at chr 12 (NAV3) after wet-lab genotyping. Supplementary Fig. 6: NAV3 expression in different EC cell lines, by (a) real time PCR and (b) western blotting. Shown are endometrial cancer cell lines ANC3A, JHUEM-14, MFE-296 and Ishikawa, as well as the non-cancerous immortalized E6E7hTERT. High exp indicates higher exposure time, whereas low exp indicates lower exposure time. Supplementary Fig. 7: (a-b) Representative images of time lapse microscopy to analyse mitotic time duration in Ishikawa and E6E7hTERT cells after silencing treatment, and (c) of green labelled cells analysed in Ishikawa cells after NAV3 overexpression. Supplementary Fig. 8: Representative images from scratch analysis in (a) Ishikawa and (b) E6E7hTERT after silencing NAV3. Supplementary Fig. 9: (a) xCELLigence impedance profile in Ishikawa cells and E6E7hTERT cells with neg. control siRNA and after silencing NAV3, N = 1 biological experiment, with 4 technical replicates. p value indicated after overall ANOVA across all time points. (b) Results from flow cytometry-based cell cycle analysis in Ishikawa cells. Values shown are % of all cells, n = 3 biological experiments, p value after unpaired t-test. (c) ATP levels measured after NAV3 silencing in Ishikawa and E6E7hTERT cells after 72H. p value indicated after paired t test. N = 4 and 3 biological experiments (respectively) with 3 technical triplicates each. (d) ATP levels measured after NAV3 overexpression in Ishikawa cells after 24H. p value indicat [file mmc14.pdf]
